# Buccolingual and Mesiodistal Dimensions of the Permanent Teeth, Their Diagnostic Value for Sex Identification, and Bolton Indices

**DOI:** 10.1155/2022/8381436

**Published:** 2022-02-10

**Authors:** Vahid Rakhshan, Fataneh Ghorbanyjavadpour, Negin Ashoori

**Affiliations:** ^1^Department of Anatomy, Dental School, Azad University of Medical Sciences, Tehran, Iran; ^2^Department of Orthodontics, School of Dentistry, Ahvaz Jundishapur University of Medical Sciences, Ahvaz, Iran; ^3^School of Dentistry, Ahvaz Jundishapur University of Medical Sciences, Ahvaz, Iran

## Abstract

**Introduction:**

We aimed (1) to measure the mesiodistal and buccolingual widths of the permanent dentition in Iranian orthodontic patients, (2) to determine cut-off points for sex identification based on the mesiodistal and buccolingual diameters, and (3) to calculate Bolton indices.

**Methods:**

The mesiodistal and buccolingual dimensions of 28 maxillary and mandibular permanent teeth in 331 Iranian nonsyndromic orthodontic patients (dental casts and radiographs) aged 12 to 35 years old with fully erupted permanent dentitions (except the third molars and some sporadic cases of a few teeth missing or excluded) were measured. The anterior, posterior, and overall Bolton ratios were calculated in cases with no missing teeth in the 6-to-6 range. Potentially associated factors (the skeletal Angle classes, crowding, sex, jaws, sides, and age), as well as the value of these measurements for sex determination and cut-off points for sex identification based on these measurements were assessed using receiver-operator characteristic (ROC) curves, analysis of variance (ANOVA), Tukey, unpaired *t*-test, partial and Pearson correlation coefficients, and multiple linear regression (*α* = 0.05).

**Results:**

Sex dimorphism was very frequent (*P* ≤ 0.05 in 41 out of 56 measurements). Only the buccolingual widths of the maxillary lateral and the mandibular central and lateral differed across the Angle classes (ANOVA/Tukey, *P* < 0.05). Cut-off points were estimated for 38 dental measurements, which were proper for sex identification (*P* < 0.05), with 8 (2 maxillary and 6 mandibular) measurements being highly appropriate (having areas under ROC curves ≥ 64%, *P* < 0.05). Both the mandibular canines were the only teeth with all four measurements highly appropriate for this purpose. Controlling for the role of sex, aging was associated negatively with several crown dimensions (the buccolingual widths of the maxillary first and second premolar and mandibular second premolar and first molar; the mesiodistal diameters of the maxillary central, canine, first premolar, and first molar, mandibular central, lateral, first premolar, and first molar, *P* ≤ 0.05, partial correlation coefficient). There were significant correlations among crown sizes. All the 28 (right/left-averaged) measurements were smaller in microdontia cases (*P* ≤ 0.002). The anterior, posterior, and overall Bolton indices were 78.05, 105.42, and 91.87, respectively. There were correlations between the overall Bolton ratio with the other two Bolton ratios (Pearson *R* = 0.696, *R* = 0.740, *P* < 0.0005) but not between the anterior and posterior Bolton ratios (*R* = 0.045, *P* = 0.459). The skeletal Angle classes might not be associated with the overall and anterior Bolton ratios (ANOVA, regression, Pearson, *P* > 0.05). However, the posterior Bolton ratio was smaller in class II cases compared to classes I or III (Tukey, *P* ≤ 0.045). In the whole sample, there was no sex dimorphism in Bolton ratios (*t*-test, *P* > 0.05). However, in Angle class II patients, the anterior Bolton ratio was greater in men than in women (*P* = 0.014).

**Conclusions:**

Sex dimorphism might be very common in the dentition of Iranians, with aging significantly reducing some measurements. The buccolingual widths of some incisors might differ across the skeletal Angle classes. Mandibular canines are the most appropriate teeth for sex identification. The Angle classes might not be associated with the anterior and overall Bolton ratios; nevertheless, the posterior Bolton ratio might be smaller in class II cases compared to others. In general, sex might not affect Bolton ratios; however, in class II patients, the anterior Bolton ratios might be larger in men.

## 1. Introduction

An important issue in dentistry is metric dental traits or mesiodistal and buccolingual crown sizes [1]. Tooth sizes are important in orthodontics, prosthodontics, restorative dentistry, anatomy, and even anthropological and forensic studies. One of the functions of orthodontists is to correct problems caused by dental size discrepancies in order to improve the mastication efficiency, the beauty of the face, and the orderliness of the dental arch [2]. Knowing the size of the teeth in populations and individuals is critical for proper diagnosis, planning an appropriate treatment, and predicting the results of orthodontic treatment [2–4]. The buccolingual dimension of the teeth is clinically important as one of the determining factors of the width of the upper and lower jaws, the width of the palate, and the space of the tongue. Therefore, the buccolingual dimensions of the teeth are related to the correct arrangement of the posterior teeth [5]. The mesiodistal dimension of the teeth has crucial orthodontic implications: to obtain an optimal occlusion, the mesiodistal measurements of the mandibular and maxillary teeth should relate to each other [6, 7]. Considerable intermaxillary mesiodistal size discrepancies—which are not uncommon—disallow aligning the teeth into an optimal occlusion [7–9]. To account for such intermaxillary relationships, Bolton [10] devised the concept of anterior and overall intermaxillary mesiodistal tooth size ratios (Bolton indices). Later, it was shown that Bolton ratios might be ethnic-specific and therefore should be assessed in different populations [6, 7, 11].

Dental crown dimensions can be used in anthropological studies, evolutionary research, and forensic sciences [3, 12–15]. Gender identification in injured bodies is an essential step and even the first step for forensic purposes [16, 17]. Determining sex through dental traits is a common practice in forensic dentistry and anthropology [18]. The most common measurements used for such purposes are mesiodistal and buccolingual widths which are convenient and reliable [19]. Numerous factors can interfere with tooth size variability, including genetic, epigenetic, or environmental factors [20]. Dental crowns might be larger in men than in women, especially in the case of the canines [13, 21–26]. Therefore, teeth are one of the desirable items for human and sex identification [24, 27, 28]. Dental sizes might also be used to estimate age [29].

Since not many studies have been done on metric dental traits especially large studies or studies in the Iranian population, we aimed to document the metric dental traits (56 mesiodistal and buccolingual crown dimensions of 28 permanent teeth) and then to determine sex dimorphism in each of the dimensions of each permanent tooth. Furthermore, the usefulness of these measurements in identifying the sex was assessed, and the cut-off point for gender determination was estimated. The associations between metric dental traits with the skeletal Angle classification and crowding were examined. Finally, we measured the Bolton intermaxillary mesiodistal tooth size ratios (Bolton indices); we also evaluated the associations between Bolton ratios with the skeletal Angle classes, sex, and age. Besides, we compared the Bolton ratios in this ethnic group with the original ratios measured by Bolton in American Caucasians [10].

## 2. Materials and Methods

This cross-sectional epidemiological study was performed on 662 maxillary and mandibular dental casts of 331 Iranian orthodontic patients attending the Orthodontic Department and two private orthodontic clinics in Ahvaz, Iran.

For data collection, all the available patients' records and their archival radiographs and casts were subsequently checked and approved/rejected until reaching the desired sample size. The inclusion criteria were being Iranian, 12 to 35 years old, and having a full permanent dentition except for the third molars and with no more than 2 extractions. The exclusion criteria were patients with cleft palates or lips or any systemic diseases or syndromes; patients with any history of previous prosthodontic, surgical, or orthodontic treatments; patients without a complete set of permanent teeth (except cases of hypodontia, cases of single excluded teeth, cases of one or two extracted teeth, and also except the third molars); cases with more than two extracted teeth; patients with more than two partially erupted permanent teeth; cases with poor cast quality; and cases without lateral cephalographs and panoramic radiographs. Additionally, single teeth that were not fully erupted or had (visible or a filed history of) dental caries, crown fractures, restorations, or veneers were excluded. Information on age, sex, and type of the skeletal Angle classification was recorded from the patients' files and their cephalographs. Data collection was performed from 2018 to 2020 [30, 31].

The used casts and radiographs were all archival, and thus, no harm was identified with this study. The protocol ethics were approved by the research committee of the university in accordance with the Helsinki Declaration (ethics code: U-98142).

All the used dental casts had been poured with white dental stone for orthodontic use. All the 56 dental buccolingual and mesiodistal dimensions of the 28 teeth were measured by a trained dentist at the quarter level (for each hemimaxilla or hemimandible of each patient separately): a digital caliper at an accuracy of 0.01 mm was used to measure the buccolingual distance (the largest distance between the buccal and lingual surfaces of the crown perpendicular to the mesiodistal width of that tooth, from the buccal to the lingual height of contours) and mesiodistal dimension (as the maximum distance between the mesial contact point and distal contact point, when the caliper is parallel to the buccal tooth surface); in case the proximal tooth was absent or the tooth was rotated, the anatomically normal contact points of the tooth would be detected by the observer [1, 20]. Microdontia was considered a very small size of a tooth but with a normal shape [32].

Cases with any missing teeth within the tooth range of bimaxillary first 12 teeth (bilateral centrals to the first molars) were identified and excluded. In the remaining 268 patients with no missing teeth in the bimaxillary 6–6 range, the sums of the mesiodistal diameters of the anterior 3 teeth (canine-to-canine) were calculated in the maxilla and also in the mandible. The anterior Bolton ratio was calculated as “100 × the sum of the mesiodistal widths of the 6 mandibular anterior teeth/the sum of the mesiodistal widths of the 6 maxillary anterior teeth” [7–10]. Similarly, in these 268 cases, the sums of the mesiodistal widths of the anterior 12 teeth (6–6) in the maxilla and also in the mandible were calculated. The overall Bolton ratio was computed as “100 × the sum of the mesiodistal diameters of the mandibular first 12 teeth (6–6, from the right first molar to the left first molar)/the sum of the mesiodistal dimensions of the maxillary first 12 teeth” [7–10]. The sums of the mesiodistal widths of the bimaxillary bilateral first premolar, second premolar, and first molar were calculated. The posterior Bolton ratio was calculated as “100 × the sum of the mesiodistal measurements of the mandibular premolars and first molars/the sum of the mesiodistal widths of the maxillary premolars and first molars” [33, 34].

### 2.1. Interexaminer Reproducibility Assessment

A second observer (FG) measured all the buccolingual and mesiodistal dimensions in all teeth of 35 randomly selected patients (4 quadrants, each). The intraclass correlation coefficient (a total of 28 Cronbach alpha values) showed excellent and high interobserver agreements between the two observers in most examinations (12 out of 28 Cronbach alpha values >0.9, 11 other Cronbach alpha values between 0.8 and 0.9, four remaining Cronbach alpha values between 0.75 and 0.8, and one last Cronbach alpha = 0.664, all *P* values < 0.0005).

### 2.2. Statistical Analyses

Statistical analysis was performed using SPSS 25 (IBM, Armonk, NY, USA). Descriptive statistics and 95% confidence intervals (CIs) were calculated. Since age might affect some crown dimensions [35], the ages of males and females were compared using an unpaired *t*-test. Crown dimensions were compared between men and women, using an unpaired *t*-test. A receiver operating characteristic (ROC) curve was used to estimate the areas under the curve (AUC) and cut-off points for the identification of individuals' sex based on dental measurements. A partial correlation coefficient, controlling for the variable sex, was used to assess correlations between age and crown measurements as well as correlations among dental measurements. In all of these analyses, the analyses for the right and left sides were conducted separately.

#### 2.2.1. Associations between Metric Traits with the Angle Classification and Crowding

The averages were calculated for measurements on the left and right sides. Associations between these average buccolingual or average mesiodistal dimensions with the skeletal Angle classes, crowding, and microdontia were assessed using an independent-sample *t*-test as well as a one-way analysis of variance (ANOVA) followed by a Tukey post hoc test.

#### 2.2.2. Bolton Anterior, Posterior, and Overall Ratios

An unpaired *t*-test and a one-way ANOVA followed by a Tukey test were used to compare the Bolton ratios between males and females and among the Angle classes, respectively. The effects of sex and the Angle classes on Bolton ratios were assessed using a multiple linear regression. Correlations between age and Bolton ratios were assessed using a Pearson correlation coefficient. The Bolton ratios were compared with the original ratios reported by Bolton [10] using an unpaired *t*-test. The level of significance was set at 0.05.

## 3. Results

There were 74 males and 257 females included in the study. The mean (SD) age of patients was 19.21 ± 4.87 years (range: 12–35). Mean ages of men and women were 18.29 ± 20.49 and 18.55 ± 19.76 years, respectively. The sexes were balanced in terms of age (*t*-test, *P* = 0.716). Of the patients, 182 (55.7%), 127 (38.8%), and 18 (5.5%) were classes I, II, and III, respectively (the Angle classifications of four patients were not entered). Crowding was observed in 89 out of 331 cases (26.9%).

Numerous teeth had sex dimorphism in terms of buccolingual or mesiodistal measurements (*t*-test, *P* values ≤ 0.05, Tables 1 and 2). The few measurements without sex dimorphism in the maxilla were as follows: mesiodistal dimensions of the lateral and both premolars on the right and the lateral and first premolars on the left. In the mandible, the sizes without sex dimorphism were as follows: the buccolingual widths of the central, lateral, and second premolars on the right, and the left lateral, as well as the mesiodistal measurements of the right central and premolars, and the left incisors and second premolar.

The *t*-test did not show any significant differences between the left versus right sides in any of the teeth of either the maxilla or the mandible (all *P* values > 0.05).

**Figure 1:**
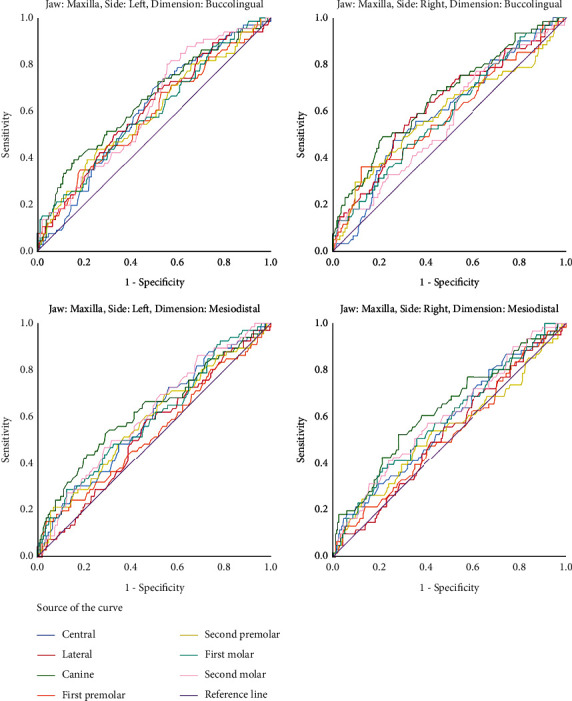
ROC curves of all the assessed mesiodistal and buccolingual dimensions of all the teeth in the left and right sides of the maxilla.

**Figure 2:**
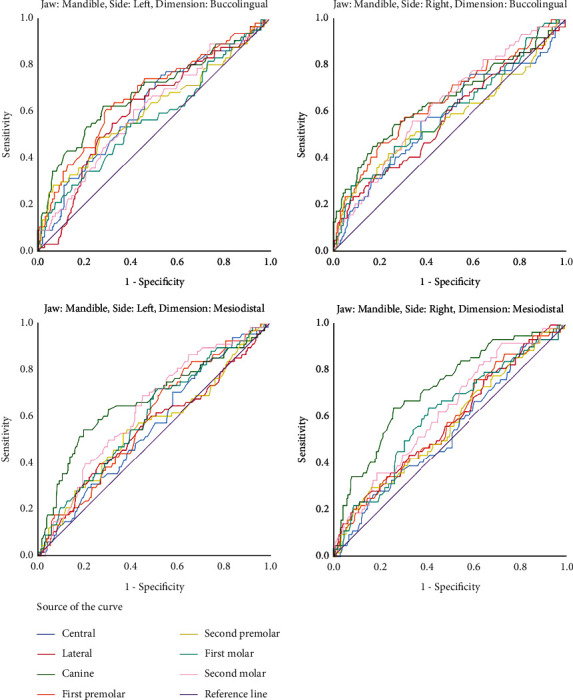
ROC curves of all the assessed mesiodistal and buccolingual dimensions of all the teeth in the left and right sides of the mandible.

The statistically significant areas under the ROC curves indicated that numerous teeth can be used for sex determination (Figure 1, Table 3) although AUCs were not considerably large in many of the statistically significant measurements. In each measurement of each quadrant, the canine had the greatest area under the curve among all other teeth. The highest AUC belonged to the mesiodistal dimension of the mandibular canine. The measurements with AUCs ≥ 64% were as follows: the buccolingual size of the right and left maxillary canines and the buccolingual size of the right and left mandibular canines and the right and left mandibular first premolars, as well as the mesiodistal dimension of the right and left mandibular canines (Figures 1 and 2, Table 3). The cut-off points for determining the sex based on the buccolingual and mesiodistal measurements of the maxillary and mandibular permanent teeth are presented in Table 3.

**Figure 3:**
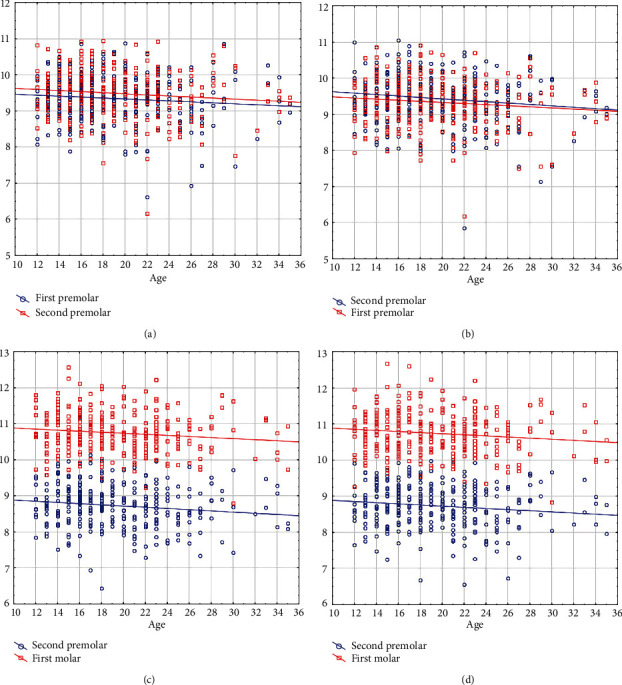
Scatterplots showing the significant correlations between age (the *X* axis, year) and the buccolingual widths (the *Y* axis, mm), in (a) the right maxillary teeth, (b) the left maxillary teeth, (c) the right mandibular teeth, and (d) the left mandibular teeth.

Controlling for the role of sex, age was negatively and weakly correlated with buccolingual widths of the right maxillary first premolar (*r* = −0.119, *P* = 0.045, partial correlation coefficient) and second premolar (*r* = −0.121, *P* = 0.040, Figure 3(a)), the left maxillary first premolar (*r* = −0.131, *P* = 0.025) and second premolar (*r* = −0.145, *P* = 0.013, Figure 3(b)), the right mandibular second premolar (*r* = −0.138, *P* = 0.017) and first molar (*r* = −0.155, *P* = 0.007, Figure 3(c)), and the left mandibular second premolar (*r* = −0.131, *P* = 0.023) and first molar (*r* = −0.135, *P* = 0.019, Figure 3(d), Appendix 1).

**Figure 4:**
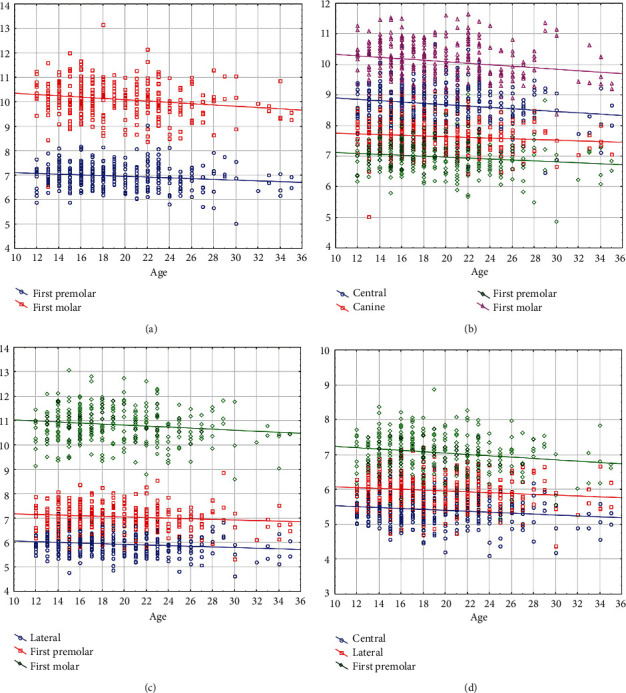
Scatterplots illustrating the significant correlations between age (the *X* axis, year) and the mesiodistal widths (the *Y* axis, mm), in (a) the right maxillary teeth, (b) the left maxillary teeth, (c) the right mandibular teeth, and (d) the left mandibular teeth.

Age was also correlated negatively, significantly, and weakly with mesiodistal dimensions of the right maxillary first premolar (*r* = −0.124, *P* = 0.034) and first molar (*r* = −0.185, *P* = 0.002, Figure 4(a)); the left maxillary central (*r* = −0.159, *P* = 0.006), canine (*r* = −0.129, *P* = 0.027), first premolar (*r* = −0.133, *P* = 0.023), and first molar (*r* = −0.134, *P* = 0.022, Figure 4(b)); the right mandibular lateral (*r* = −0.177, *P* = 0.002), first premolar (*r* = −0.149, *P* = 0.010); and first molar (*r* = −0.159, *P* = 0.006, Figure 4(c)); and the left mandibular central (*r* = −0.163, *P* = 0.004), lateral (*r* = −0.131, *P* = 0.022), and first premolar (*r* = −0.175, *P* = 0.002, Figure 4(d), Appendix 1).

### 3.1. Associations between Metric Traits with the Angle Classification

According to the ANOVA, the teeth that had different sizes in different classes were the maxillary lateral (buccolingual measurement only) and the mandibular central and lateral (buccolingual only, Table 4). According to the Tukey post hoc test, the buccolingual dimension of maxillary lateral differed only between classes I and II (*P* = 0.030). Similarly, the buccolingual width of the mandibular central differed only between classes I and II (*P* = 0.032). The buccolingual diameter of the mandibular lateral differed between classes I and II (*P* = 0.025, Table 4).

All dental measurements were similar between cases with and without crowding (*t*-test, *P* > 0.05, Table 5).

All “left/right-averaged” buccolingual and mesiodistal measurements of all the 14 teeth (the maxillary and mandibular centrals to the second molars, regardless of their right and left sides) differed significantly between the cases with microdontia versus those without it (*t*-test, *P* ≤ 0.002, Table 6).

There were significant positive correlations among all different crown measurements of all the assessed teeth (Appendix 1).

### 3.2. Bolton Indices

Between men and women, there was no significant difference in terms of Bolton ratios (*t*-test, Table 7). There was no significant difference among different Angle classes in terms of the overall or anterior Bolton ratios (Table 7). However, the posterior Bolton ratios differed significantly across the Angle classes (ANOVA, Table 7). The Tukey test showed that the mean posterior Bolton ratio in class II patients was smaller than those in both class I (*P* = 0.029) and class III patients (*P* = 0.045). There was no significant difference between classes I and III (*P* = 0.369, Tukey). The multiple regression did not detect any significant effect of sex (*P* ≥ 0.080) or the Angle classification (*P* ≥ 0.304) on any Bolton ratios.

There was no correlation between ages with any Bolton ratios (Pearson *R* ≤ 0.064, *P* ≥ 0.297). The correlations between the overall Bolton index with the anterior Bolton index (Pearson *R* = 0.696, *P* < 0.00000005) and the posterior Bolton index (*R* = 0.740, *P* < 0.00000005) were significant. However, the was no significant correlation between the anterior and posterior Bolton ratios (*R* = 0.045, *P* = 0.459).

The unpaired *t*-test was used to compare the sexes within each Angle class separately (Table 8). Because of the small number of class III males, no comparisons were done for class III cases. As the only significant comparison, the anterior Bolton ratio of class II men was significantly larger than that of class II women (*P* = 0.014, Table 8).

The comparison of the overall Bolton ratio of this sample (Table 7) with the original overall Bolton ratio (mean: 91.3, SD: 1.91, *n* = 55) [10] did not show a significant difference (unpaired *t*-test, *P* = 0.107). However, the anterior Bolton ratio of this sample was significantly greater (*t*-test, *P* = 0.0498, Table 7) than the original anterior Bolton ratio (mean: 77.2, SD: 1.65, *n* = 55) [10].

## 4. Discussion

Tooth size variation is influenced by environmental and genetic factors including race, sex, heredity, cellular changes, and bilateral asymmetry [4, 20, 36]. Environmental factors include nutrition, disease, and climate, which might affect the prenatal dental system and seem to make little change to the normal dental system [37]. The strong contribution of genetic factors to the differences in dental measurements has been shown, but the influence of environmental factors seems plausible as well. Both environmental and genetic factors play a role in the etiology of supernumerary teeth, hypodontia, megadontia, and microdontia [38]. Sizes of teeth might vary in different populations [1, 20, 39]. Sex dimorphism has been reported as ranging between 0.82% and 5.97% for all teeth [4]. An example of a sex difference is the tendency of men to have larger teeth than women, which reflects the relationship between the X chromosome and the Y chromosome. For example, men who are XXY and XYY have teeth larger than XY men [1]. Our results were in line with these suggestions.

Keiser and Julius examined mesiodistal and buccolingual tooth sizes and concluded that they could be used to determine sex [40]. Using the dental dimensions of one ethnic group might be used in other ethnicities as well [41]. One of the preferred methods is to use the canine index, which uses the mesiodistal size of the mandibular canine together with intercanine width [42–44]. But the most widely used method is the mesiodistal and buccolingual dimensions [13, 22, 23]. The mandibular canine seems to have the greatest sex dimorphism among all teeth while incisors might have the least sexual dimorphism [24–26]. A recent meta-analysis suggested that the canine might have the most sex dimorphism among all teeth, which might be due to the longer duration of amelogenesis of this tooth in men compared to women [4]. This is in agreement with our findings of the possibility of the use of mandibular canines in predicting gender. Some researchers have shown that when the mesiodistal size of the canine tooth is larger than 7.0 to 7.2 mm, there is a very high probability that the person is male [16, 24, 45], and this was in line with our results pertaining to the mandibular canine. Some authors have suggested that both the mesiodistal and buccolingual dimensions are needed together for sex determination [46]. In our study, many molar teeth could be used for sex identification. In earlier research, this tooth was sometimes useful, and in some studies, it was useful merely alongside other teeth for sex determination, indicating the role of ethnicity in sex dimorphism [47–51].

Our findings indicated that aging might reduce the mesiodistal and buccolingual dimensions of certain teeth. In archaeological studies, the pattern of increased wear appears to be age-dependent, while in modern populations, men are more prone to tooth wear than women [35]. Such wear might affect both epidemiological and clinical outcomes and should be taken into account in such examinations.

The Bolton ratios found in this study were within the range reported earlier [6, 7, 11, 33, 34]. In comparison to the original Bolton ratios, our sample's anterior Bolton ratio was larger. This should be considered when practicing on Iranian patients; still, it should be noted that such results are not definitive, and sometimes, even studies conducted within the same ethnicity and country yield different results [6, 11]. The Angle classes were not associated with the anterior and posterior Bolton ratios in this sample. This finding was similar to some previous studies [8, 52–54] but in contrast to some others [33, 55]. It was found, however, that the posterior Bolton ratio might be smaller in class II patients, compared to classes I and III. In terms of sex dimorphism in Bolton ratios, when our whole sample was assessed, no sexual dimorphism was observed in this study. This finding was in line with most previous studies as well as the conclusion of a recent meta-analysis on Bolton ratios [6, 7, 53–55]. However, when sex dimorphism was examined separately within each of the Angle classes I or II, it was found that in class II patients, the anterior Bolton ratio might be greater in men than in women. We observed a 70% positive correlation between the anterior and overall Bolton indices. This was greater than the studies of Bolton (50% correlation) [10] or White (-12% correlation) [56] but slightly smaller than a study on Sudanese people (79% correlation) [7]. The controversies might be attributable to real ethnic differences as well as methodological variations such as eligibility criteria or sample sizes. The concept of the posterior Bolton ratio is introduced and assessed in merely two studies [33, 34]. We observed a 74% correlation between the posterior and overall Bolton ratios and almost no correlation between the anterior and posterior Bolton ratios. More studies are needed on the posterior Bolton ratio.

This study was limited by some factors. The number of females was much greater than males, although both seemed to be adequate. Moreover, the sample size pertaining to the Bolton ratios of class III men was very small. Hence, we did not perform inferential statistics on this subgroup. The generalizability of some aspects of this research was limited to the target population (Iranian orthodontic patients).

## 5. Conclusions

Within the limitations of this study, the following key points can be summarized:

Sex dimorphism existed in most dental measurements. ROC curve analyses indicated that (A) the mandibular teeth mostly seemed better than the maxillary ones for sex identification; (B) the most appropriate dental measurements for sex determination were the buccolingual dimension of the right and left maxillary canines, the buccolingual measurement of the right and left mandibular canines and the right and left mandibular first premolars, as well as (C) the mesiodistal dimension of the right and left mandibular canines
Cut-off points for sex identification based on proper dental measurements were calculated for 38 teeth. In the maxilla, the buccolingual cut-off points ranged from 7.715 mm for the central to 11.715 mm for the first molar; the mesiodistal cut-offs ranged from 8.750 mm for the central to 10.815 mm for the first molar. In the mandible, the range of buccolingual cut-off points was 6.175 mm to 11.455 mm (the central to the first molar), while the range of mesiodistal cut-off points was 6.835 mm to 10.910 mm (the canine to the first molar).
(A) Aging might slightly reduce the buccolingual crown dimension in a few posterior teeth: the right and left maxillary first premolar and second premolar and right and left mandibular second premolar and first molar. (B) It might also slightly reduce the mesiodistal widths of certain anterior and posterior teeth: the right maxillary first premolar and first molar, the left maxillary central, canine, first premolar, first molar, the right mandibular lateral, first premolar, and first molar, and the left mandibular central, lateral, and first premolar
(A) The only measurements differing among the skeletal Angle classes were the buccolingual widths of the maxillary lateral, the mandibular central, and the mandibular lateral. These differed mainly between classes I and II. (B) Dental measurements might not differ between crowded and noncrowded dentitions. (C) All crown sizes might be smaller in microdontia cases compared to cases without this anomaly
The anterior, posterior, and overall Bolton indices were 78.05, 105.42, and 91.87, respectively. The skeletal Angle classification might not be associated with the anterior and overall Bolton ratios. However, class II patients might have smaller posterior Bolton ratios compared to class I or III patients. Aging might not affect Bolton indices. In the whole sample, there was no sexual dimorphism in either of these indices. However, in class II patients, the anterior Bolton ratio was greater in men than in women. There were 69.6% and 74.0% correlations between the overall Bolton indexes with the anterior and posterior Bolton indices, respectively. The anterior and posterior Bolton indices might not be correlated. The overall Bolton ratio in this population might not differ much from the original overall Bolton ratio. Nonetheless, this population's anterior Bolton ratio might be greater than Bolton's original anterior ratio

## Figures and Tables

**Table 1:** Descriptive statistics and 95% CIs for the mesiodistal and buccolingual widths (mm) in the right and left sides of the maxilla in males versus females (compared using the *t*-test).

Side	Dimension	Tooth	Sex	*N*	Mean	SD	95% CI		Min	Max	*P*
Right	Buccolingual	1	Female	257	7.369	0.584	7.30	7.44	5.61	9.22	0.044
			Male	73	7.522	0.520	7.40	7.64	6.34	8.88	
			Total	330	7.403	0.573	7.34	7.47	5.61	9.22	
		2	Female	250	6.536	0.644	6.46	6.62	4.47	8.15	0.003
			Male	72	6.812	0.816	6.62	7.00	4.22	9.91	
			Total	322	6.598	0.694	6.52	6.67	4.22	9.91	
		3	Female	253	8.178	0.647	8.10	8.26	5.65	9.65	<0.0005
			Male	73	8.497	0.751	8.32	8.67	6.36	9.88	
			Total	326	8.250	0.683	8.18	8.32	5.65	9.88	
		4	Female	248	9.302	0.642	9.22	9.38	6.62	10.87	0.031
			Male	72	9.488	0.624	9.34	9.63	7.79	10.85	
			Total	320	9.344	0.642	9.27	9.41	6.62	10.87	
		5	Female	254	9.450	0.644	9.37	9.53	6.16	10.94	0.047
			Male	73	9.622	0.657	9.47	9.77	8.29	10.92	
			Total	327	9.489	0.650	9.42	9.56	6.16	10.94	
		6	Female	254	11.373	0.631	11.30	11.45	9.58	12.78	0.008
			Male	72	11.600	0.640	11.45	11.75	10.24	13.11	
			Total	326	11.423	0.639	11.35	11.49	9.58	13.11	
		7	Female	253	11.338	0.798	11.24	11.44	8.75	13.60	0.033
			Male	70	11.570	0.817	11.38	11.76	9.19	13.26	
			Total	323	11.388	0.806	11.30	11.48	8.75	13.60	
	Mesiodistal	1	Female	257	8.626	0.573	8.56	8.70	6.60	10.37	0.031
			Male	73	8.789	0.556	8.66	8.92	7.62	9.98	
			Total	330	8.662	0.572	8.60	8.72	6.60	10.37	
		2	Female	250	6.759	0.708	6.67	6.85	3.90	9.69	0.560
			Male	72	6.812	0.620	6.67	6.96	4.70	8.14	
			Total	322	6.771	0.689	6.70	6.85	3.90	9.69	
		3	Female	255	7.675	0.467	7.62	7.73	6.12	9.15	0.001
			Male	73	7.896	0.509	7.78	8.02	6.93	8.82	
			Total	328	7.724	0.485	7.67	7.78	6.12	9.15	
		4	Female	249	6.946	0.499	6.88	7.01	5.02	9.04	0.187
			Male	72	7.034	0.497	6.92	7.15	5.67	8.16	
			Total	321	6.966	0.499	6.91	7.02	5.02	9.04	
		5	Female	254	6.665	0.499	6.60	6.73	4.93	9.08	0.472
			Male	73	6.713	0.513	6.59	6.83	5.78	8.05	
			Total	327	6.676	0.502	6.62	6.73	4.93	9.08	
		6	Female	254	10.050	0.723	9.96	10.14	6.54	13.16	0.018
			Male	72	10.277	0.691	10.11	10.44	8.52	12.13	
			Total	326	10.100	0.721	10.02	10.18	6.54	13.16	
		7	Female	252	9.780	0.676	9.70	9.86	7.60	11.63	0.001
			Male	70	10.076	0.616	9.93	10.22	8.68	11.45	
			Total	322	9.844	0.674	9.77	9.92	7.60	11.63	

Left	Buccolingual	1	Female	256	7.342	0.620	7.27	7.42	5.79	9.01	0.017
			Male	74	7.536	0.591	7.40	7.67	5.78	9.57	
			Total	330	7.386	0.618	7.32	7.45	5.78	9.57	
		2	Female	252	6.547	0.627	6.47	6.62	4.62	8.41	0.013
			Male	72	6.782	0.931	6.56	7.00	4.80	12.46	
			Total	324	6.599	0.711	6.52	6.68	4.62	12.46	
		3	Female	250	8.172	0.694	8.09	8.26	5.85	9.87	<0.0005
			Male	73	8.500	0.727	8.33	8.67	6.20	10.05	
			Total	323	8.246	0.714	8.17	8.32	5.85	10.05	
		4	Female	250	9.297	0.633	9.22	9.38	6.19	10.91	0.026
			Male	72	9.485	0.616	9.34	9.63	8.27	10.86	
			Total	322	9.339	0.633	9.27	9.41	6.19	10.91	
		5	Female	253	9.406	0.675	9.32	9.49	5.86	10.90	0.040
			Male	74	9.588	0.641	9.44	9.74	8.18	11.05	
			Total	327	9.447	0.670	9.37	9.52	5.86	11.05	
		6	Female	256	11.327	0.629	11.25	11.40	9.53	12.94	0.004
			Male	74	11.568	0.653	11.42	11.72	10.03	13.24	
			Total	330	11.381	0.641	11.31	11.45	9.53	13.24	
		7	Female	253	11.247	0.779	11.15	11.34	8.35	13.14	0.001
			Male	71	11.602	0.727	11.43	11.77	9.61	13.62	
			Total	324	11.325	0.781	11.24	11.41	8.35	13.62	
	Mesiodistal	1	Female	256	8.659	0.607	8.58	8.73	6.46	10.61	0.017
			Male	74	8.851	0.601	8.71	8.99	7.52	10.67	
			Total	330	8.702	0.610	8.64	8.77	6.46	10.67	
		2	Female	252	6.788	0.636	6.71	6.87	4.57	8.43	0.685
			Male	72	6.822	0.624	6.68	6.97	4.64	7.96	
			Total	324	6.795	0.633	6.73	6.86	4.57	8.43	
		3	Female	251	7.602	0.478	7.54	7.66	5.02	9.13	0.001
			Male	73	7.814	0.527	7.69	7.94	6.66	9.02	
			Total	324	7.650	0.496	7.60	7.70	5.02	9.13	
		4	Female	250	6.965	0.503	6.90	7.03	4.86	9.02	0.280
			Male	72	7.039	0.543	6.91	7.17	5.49	8.38	
			Total	322	6.981	0.512	6.93	7.04	4.86	9.02	
		5	Female	253	6.642	0.575	6.57	6.71	5.01	9.73	0.037
			Male	74	6.805	0.646	6.66	6.95	5.61	9.96	
			Total	327	6.679	0.595	6.61	6.74	5.01	9.96	
		6	Female	256	10.059	0.632	9.98	10.14	8.26	11.53	0.005
			Male	74	10.297	0.642	10.15	10.45	8.96	11.65	
			Total	330	10.113	0.641	10.04	10.18	8.26	11.65	
		7	Female	253	9.845	0.648	9.77	9.93	8.20	11.66	0.002
			Male	71	10.115	0.561	9.98	10.25	8.95	11.43	
			Total	324	9.904	0.639	9.83	9.97	8.20	11.66	

Tooth numbers 1 to 7 denote the most anterior (the central) to the most posterior (the second molar) teeth. SD: standard deviation; CI: confidence interval; Min: minimum; Max: maximum.

**Table 2:** Descriptive statistics and 95% CIs for crown measurements (mm) in the mandible, compared between the sexes (using the *t*-test).

Side	Dimension	Tooth	Sex	*N*	Mean	SD	95% CI		Min	Max	*P*
Right	Buccolingual	1	Female	255	6.243	0.489	6.18	6.30	4.50	8.02	0.104
			Male	73	6.352	0.546	6.22	6.48	4.95	7.63	
			Total	328	6.267	0.503	6.21	6.32	4.50	8.02	
		2	Female	255	6.511	0.487	6.45	6.57	5.26	8.33	0.078
			Male	74	6.634	0.651	6.48	6.78	4.79	7.89	
			Total	329	6.539	0.530	6.48	6.60	4.79	8.33	
		3	Female	256	7.398	0.589	7.33	7.47	5.63	8.75	<0.0005
			Male	73	7.783	0.799	7.60	7.97	5.80	9.65	
			Total	329	7.483	0.660	7.41	7.55	5.63	9.65	
		4	Female	253	7.956	0.587	7.88	8.03	6.00	9.50	<0.0005
			Male	73	8.258	0.660	8.10	8.41	7.05	9.97	
			Total	326	8.024	0.616	7.96	8.09	6.00	9.97	
		5	Female	250	8.700	0.585	8.63	8.77	6.44	11.18	0.085
			Male	69	8.841	0.651	8.68	9.00	7.60	9.96	
			Total	319	8.731	0.601	8.66	8.80	6.44	11.18	
		6	Female	254	10.704	0.530	10.64	10.77	8.80	12.22	0.002
			Male	72	10.928	0.610	10.78	11.07	9.74	12.56	
			Total	326	10.753	0.556	10.69	10.81	8.80	12.56	
		7	Female	255	10.509	0.635	10.43	10.59	8.56	12.29	<0.0005
			Male	71	10.822	0.608	10.68	10.97	9.36	12.19	
			Total	326	10.577	0.641	10.51	10.65	8.56	12.29	
	Mesiodistal	1	Female	257	5.380	0.445	5.33	5.43	4.08	6.80	0.377
			Male	73	5.431	0.391	5.34	5.52	4.51	6.32	
			Total	330	5.391	0.433	5.34	5.44	4.08	6.80	
		2	Female	255	5.913	0.424	5.86	5.96	4.62	7.22	0.023
			Male	74	6.041	0.430	5.94	6.14	5.33	7.18	
			Total	329	5.941	0.428	5.90	5.99	4.62	7.22	
		3	Female	257	6.599	0.476	6.54	6.66	5.17	8.78	<0.0005
			Male	73	6.941	0.458	6.83	7.05	5.74	8.02	
			Total	330	6.674	0.493	6.62	6.73	5.17	8.78	
		4	Female	253	7.038	0.497	6.98	7.10	5.32	8.87	0.106
			Male	73	7.144	0.482	7.03	7.26	5.73	8.26	
			Total	326	7.061	0.495	7.01	7.12	5.32	8.87	
		5	Female	250	7.051	0.550	6.98	7.12	5.86	9.40	0.078
			Male	69	7.184	0.557	7.05	7.32	5.86	8.38	
			Total	319	7.080	0.553	7.02	7.14	5.86	9.40	
		6	Female	254	10.782	0.699	10.70	10.87	8.60	12.82	0.017
			Male	72	11.009	0.747	10.83	11.18	9.13	13.05	
			Total	326	10.832	0.715	10.75	10.91	8.60	13.05	
		7	Female	255	10.226	0.676	10.14	10.31	8.20	12.42	<0.0005
			Male	71	10.554	0.697	10.39	10.72	8.94	12.45	
			Total	326	10.297	0.693	10.22	10.37	8.20	12.45	

Left	Buccolingual	1	Female	255	6.224	0.491	6.16	6.28	4.83	7.77	0.036
			Male	74	6.364	0.548	6.24	6.49	4.57	7.70	
			Total	329	6.256	0.507	6.20	6.31	4.57	7.77	
		2	Female	256	6.487	0.544	6.42	6.55	4.88	8.20	0.081
			Male	74	6.611	0.501	6.49	6.73	5.29	8.04	
			Total	330	6.515	0.536	6.46	6.57	4.88	8.20	
		3	Female	257	7.424	0.604	7.35	7.50	5.53	9.10	<0.0005
			Male	74	7.795	0.760	7.62	7.97	6.15	9.20	
			Total	331	7.507	0.659	7.44	7.58	5.53	9.20	
		4	Female	254	7.972	0.573	7.90	8.04	6.16	9.48	<0.0005
			Male	73	8.274	0.652	8.12	8.43	6.56	9.62	
			Total	327	8.040	0.604	7.97	8.11	6.16	9.62	
		5	Female	250	8.699	0.575	8.63	8.77	6.56	10.78	0.048
			Male	71	8.860	0.682	8.70	9.02	6.73	9.96	
			Total	321	8.735	0.603	8.67	8.80	6.56	10.78	
		6	Female	250	10.707	0.558	10.64	10.78	8.83	12.23	0.020
			Male	73	10.887	0.643	10.74	11.04	9.74	12.67	
			Total	323	10.748	0.582	10.68	10.81	8.83	12.67	
		7	Female	255	10.492	0.625	10.41	10.57	8.56	12.05	0.004
			Male	70	10.735	0.627	10.59	10.88	9.31	12.17	
			Total	325	10.544	0.632	10.48	10.61	8.56	12.17	
	Mesiodistal	1	Female	257	5.397	0.425	5.35	5.45	4.09	6.59	0.202
			Male	74	5.468	0.388	5.38	5.56	4.20	6.39	
			Total	331	5.413	0.417	5.37	5.46	4.09	6.59	
		2	Female	256	5.954	0.449	5.90	6.01	4.38	7.21	0.149
			Male	74	6.040	0.465	5.93	6.15	4.87	6.98	
			Total	330	5.973	0.454	5.92	6.02	4.38	7.21	
		3	Female	257	6.651	0.457	6.59	6.71	5.44	9.66	<0.0005
			Male	74	6.901	0.488	6.79	7.01	5.70	7.85	
			Total	331	6.707	0.475	6.66	6.76	5.44	9.66	
		4	Female	254	7.031	0.532	6.96	7.10	5.17	8.88	0.048
			Male	73	7.166	0.443	7.06	7.27	6.15	8.26	
			Total	327	7.061	0.516	7.00	7.12	5.17	8.88	
		5	Female	251	7.130	0.595	7.06	7.20	5.82	10.75	0.185
			Male	72	7.234	0.559	7.10	7.37	6.22	8.96	
			Total	323	7.153	0.588	7.09	7.22	5.82	10.75	
		6	Female	250	10.795	0.704	10.71	10.88	6.84	12.65	0.009
			Male	73	11.040	0.685	10.88	11.20	9.35	12.92	
			Total	323	10.851	0.706	10.77	10.93	6.84	12.92	
		7	Female	255	10.239	0.703	10.15	10.33	8.44	12.28	0.003
			Male	70	10.514	0.608	10.37	10.66	9.02	11.78	
			Total	325	10.298	0.692	10.22	10.37	8.44	12.28	

Tooth numbers 1 to 7 indicate the most anterior to the most posterior teeth. SD: standard deviation; CI: confidence interval; Min: minimum; Max: maximum.

**Table 3:** The areas under ROC curves and the cut-off points for sex determination (mm).

Jaw	Side	Dimension	Tooth	Area	SE	*P*	95% CI		Cut-off (mm)
Maxilla	Right	Buccolingual	Central	0.596	0.040	0.021	0.517	0.675	7.715
			Lateral	0.628	0.041	0.002	0.547	0.709	6.950
			Canine	0.662	0.040	<0.0005	0.584	0.741	8.665
			First premolar	0.587	0.043	0.037	0.502	0.672	9.915
			Second premolar	0.590	0.045	0.031	0.502	0.677	10.175
			First molar	0.589	0.041	0.032	0.509	0.670	11.715
			Second molar	0.567	0.041	0.107	0.487	0.648	—
		Mesiodistal	Central	0.575	0.041	0.072	0.494	0.656	—
			Lateral	0.526	0.041	0.527	0.446	0.606	—
			Canine	0.628	0.041	0.002	0.547	0.710	7.930
			First premolar	0.526	0.042	0.528	0.444	0.609	—
			Second premolar	0.536	0.044	0.383	0.449	0.623	—
			First molar	0.590	0.041	0.030	0.509	0.671	10.275
			Second molar	0.598	0.041	0.018	0.518	0.678	10.235
	Left	Buccolingual	Central	0.602	0.038	0.011	0.529	0.676	7.355
			Lateral	0.606	0.039	0.009	0.529	0.683	6.535
			Canine	0.643	0.040	<0.0005	0.566	0.721	8.780
			First premolar	0.583	0.041	0.039	0.503	0.663	9.845
			Second premolar	0.591	0.041	0.024	0.512	0.671	9.865
			First molar	0.596	0.040	0.018	0.518	0.674	11.505
			Second molar	0.616	0.037	0.004	0.543	0.690	11.235
		Mesiodistal	Central	0.591	0.039	0.025	0.513	0.668	8.570
			Lateral	0.537	0.039	0.363	0.460	0.614	—
			Canine	0.627	0.041	0.002	0.546	0.708	7.835
			First premolar	0.532	0.042	0.422	0.450	0.615	—
			Second premolar	0.583	0.041	0.041	0.503	0.663	7.365
			First molar	0.595	0.040	0.019	0.516	0.673	10.815
			Second molar	0.611	0.039	0.006	0.536	0.687	10.155

Mandible	Right	Buccolingual	Central	0.578	0.043	0.056	0.494	0.662	—
			Lateral	0.572	0.043	0.078	0.488	0.655	—
			Canine	0.652	0.043	<0.0005	0.568	0.736	7.905
			First premolar	0.652	0.041	<0.0005	0.572	0.732	8.285
			Second premolar	0.572	0.045	0.078	0.484	0.660	—
			First molar	0.594	0.042	0.021	0.512	0.677	11.455
			Second molar	0.629	0.039	0.002	0.553	0.705	10.755
		Mesiodistal	Central	0.536	0.040	0.372	0.457	0.615	—
			Lateral	0.577	0.041	0.059	0.497	0.656	—
			Canine	0.720	0.036	<0.0005	0.650	0.790	6.835
			First premolar	0.575	0.040	0.066	0.497	0.653	—
			Second premolar	0.563	0.041	0.119	0.483	0.644	—
			First molar	0.605	0.040	0.010	0.526	0.684	10.885
			Second molar	0.620	0.038	0.003	0.545	0.695	10.275
	Left	Buccolingual	Central	0.617	0.039	0.003	0.540	0.695	6.175
			Lateral	0.610	0.039	0.006	0.534	0.686	6.575
			Canine	0.683	0.041	<0.0005	0.604	0.763	7.765
			First premolar	0.673	0.039	<0.0005	0.596	0.749	8.275
			Second premolar	0.606	0.043	0.008	0.522	0.689	9.025
			First molar	0.571	0.042	0.077	0.489	0.652	—
			Second molar	0.603	0.040	0.010	0.526	0.681	10.610
		Mesiodistal	Central	0.553	0.038	0.183	0.478	0.628	—
			Lateral	0.547	0.042	0.238	0.465	0.629	—
			Canine	0.668	0.041	<0.0005	0.589	0.748	6.960
			First premolar	0.585	0.038	0.033	0.511	0.658	6.965
			Second premolar	0.554	0.041	0.172	0.473	0.635	—
			First molar	0.598	0.039	0.014	0.522	0.673	10.910
			Second molar	0.631	0.037	0.001	0.559	0.703	10.275

SE: standard error; CI: confidence interval for the AUC. Measurements below the cut-off points belong to women.

**Table 4:** Descriptive statistics and 95% CIs for dental measurements (averages of the right and left sides, mm) in different Angle classes. The classes are compared using the one-way ANOVA.

Jaw	Measurement	Tooth	Class	*N*	Mean	SD	95% CI		Min	Max	*P*
Maxilla	Buccolingual	1	I	182	7.355	0.540	7.28	7.43	5.91	8.68	0.271
			II	127	7.440	0.623	7.33	7.55	5.88	9.04	
			III	18	7.528	0.539	7.26	7.80	6.47	8.45	
		2	I	177	6.516	0.617	6.42	6.61	4.47	8.29	0.039
			II	127	6.706	0.683	6.59	6.83	5.06	10.13	
			III	17	6.635	0.576	6.34	6.93	5.84	7.66	
		3	I	179	8.193	0.657	8.10	8.29	6.24	9.62	0.205
			II	125	8.329	0.689	8.21	8.45	6.18	9.87	
			III	18	8.206	0.571	7.92	8.49	7.10	9.37	
		4	I	177	9.309	0.588	9.22	9.40	6.41	10.74	0.454
			II	125	9.397	0.646	9.28	9.51	7.50	10.69	
			III	17	9.381	0.539	9.10	9.66	8.21	10.12	
		5	I	181	9.425	0.652	9.33	9.52	6.01	10.91	0.360
			II	126	9.522	0.615	9.41	9.63	7.66	10.99	
			III	18	9.549	0.557	9.27	9.83	8.19	10.41	
		6	I	181	11.386	0.575	11.30	11.47	9.73	12.80	0.797
			II	127	11.431	0.657	11.32	11.55	9.81	13.17	
			III	18	11.440	0.689	11.10	11.78	9.96	12.78	
		7	I	178	11.336	0.752	11.23	11.45	8.98	13.04	0.651
			II	125	11.404	0.801	11.26	11.55	9.34	13.32	
			III	18	11.263	0.743	10.89	11.63	9.78	12.80	
	Mesiodistal	1	I	182	8.694	0.545	8.61	8.77	6.64	10.06	0.842
			II	127	8.656	0.580	8.55	8.76	6.84	10.47	
			III	18	8.664	0.673	8.33	9.00	7.14	9.85	
		2	I	177	6.790	0.620	6.70	6.88	3.90	8.10	0.846
			II	127	6.749	0.662	6.63	6.87	4.42	8.04	
			III	17	6.741	0.676	6.39	7.09	5.29	7.70	
		3	I	180	7.697	0.501	7.62	7.77	5.57	9.14	0.558
			II	126	7.689	0.427	7.61	7.76	6.84	8.84	
			III	18	7.572	0.386	7.38	7.76	6.78	8.10	
		4	I	177	6.984	0.488	6.91	7.06	5.58	9.03	0.849
			II	125	6.956	0.491	6.87	7.04	4.94	8.29	
			III	17	7.006	0.385	6.81	7.20	6.38	7.81	
		5	I	181	6.675	0.526	6.60	6.75	5.61	8.96	0.851
			II	126	6.673	0.484	6.59	6.76	4.97	8.09	
			III	18	6.745	0.547	6.47	7.02	5.25	7.52	
		6	I	181	10.080	0.598	9.99	10.17	8.46	11.89	0.161
			II	127	10.185	0.668	10.07	10.30	8.63	11.76	
			III	18	9.932	0.656	9.61	10.26	9.16	11.12	
		7	I	178	9.848	0.592	9.76	9.94	8.26	11.48	0.159
			II	125	9.937	0.631	9.83	10.05	8.23	11.62	
			III	18	9.666	0.688	9.32	10.01	8.77	10.98	

Mandible	Buccolingual	1	I	182	6.209	0.457	6.14	6.28	4.78	7.55	0.042
			II	125	6.348	0.491	6.26	6.43	5.20	7.82	
			III	18	6.273	0.477	6.04	6.51	5.25	7.01	
		2	I	182	6.481	0.476	6.41	6.55	5.17	7.66	0.009
			II	127	6.628	0.502	6.54	6.72	5.38	7.97	
			III	18	6.346	0.483	6.11	6.59	5.64	7.23	
		3	I	182	7.442	0.626	7.35	7.53	5.89	8.92	0.235
			II	127	7.565	0.621	7.46	7.67	6.07	9.43	
			III	18	7.480	0.638	7.16	7.80	6.42	8.75	
		4	I	178	8.002	0.572	7.92	8.09	6.11	9.59	0.484
			II	127	8.083	0.603	7.98	8.19	6.11	9.71	
			III	18	8.015	0.558	7.74	8.29	7.05	8.94	
		5	I	180	8.729	0.569	8.65	8.81	6.56	9.95	0.766
			II	124	8.747	0.596	8.64	8.85	6.56	10.18	
			III	18	8.642	0.465	8.41	8.87	7.52	9.29	
		6	I	182	10.734	0.526	10.66	10.81	9.32	12.09	0.908
			II	126	10.752	0.580	10.65	10.85	8.82	12.62	
			III	18	10.787	0.532	10.52	11.05	9.86	11.85	
		7	I	181	10.560	0.616	10.47	10.65	9.26	12.04	0.934
			II	125	10.546	0.612	10.44	10.65	8.56	12.04	
			III	18	10.600	0.546	10.33	10.87	9.56	11.62	
	Mesiodistal	1	I	182	5.391	0.393	5.33	5.45	4.12	6.41	0.814
			II	127	5.408	0.412	5.34	5.48	4.24	6.45	
			III	18	5.449	0.423	5.24	5.66	4.46	6.00	
		2	I	182	5.962	0.407	5.90	6.02	4.97	7.22	0.554
			II	127	5.959	0.434	5.88	6.04	4.50	7.11	
			III	18	5.850	0.441	5.63	6.07	4.94	6.51	
		3	I	182	6.715	0.426	6.65	6.78	5.48	8.00	0.527
			II	127	6.661	0.481	6.58	6.75	5.39	7.85	
			III	18	6.649	0.394	6.45	6.84	5.80	7.37	
		4	I	178	7.081	0.462	7.01	7.15	5.98	8.30	0.711
			II	127	7.035	0.494	6.95	7.12	5.44	8.26	
			III	18	7.051	0.559	6.77	7.33	5.72	7.57	
		5	I	181	7.127	0.498	7.05	7.20	6.06	8.79	0.656
			II	124	7.090	0.539	6.99	7.19	5.86	8.67	
			III	18	7.199	0.568	6.92	7.48	6.00	8.46	
		6	I	182	10.853	0.676	10.75	10.95	8.95	12.65	0.255
			II	126	10.769	0.723	10.64	10.90	8.17	12.99	
			III	18	11.031	0.514	10.77	11.29	10.14	11.84	
		7	I	181	10.317	0.595	10.23	10.40	8.96	12.01	0.648
			II	125	10.253	0.692	10.13	10.38	8.52	12.31	
			III	18	10.346	0.628	10.03	10.66	9.48	11.78	

Tooth numbers 1 to 7 indicate the central to the second molar teeth. SD: standard deviation; CI: confidence interval; Min: minimum; Max: maximum.

**Table 5:** Descriptive statistics and 95% CIs for dental measurements (averages of the right and left sides, mm) in crowded versus noncrowded dentitions. The groups are compared using the *t*-test.

Jaw	Measurement	Tooth	Crowding	*N*	Mean	SD	95% CI		Min	Max	*P*
Maxilla	Buccolingual	1	No	242	7.411	0.606	7.33	7.49	5.88	9.04	0.443
			Yes	89	7.356	0.480	7.26	7.46	5.93	8.17	
			Total	331	7.396	0.575	7.33	7.46	5.88	9.04	
		2	No	237	6.622	0.682	6.53	6.71	4.47	10.13	0.191
			Yes	88	6.515	0.569	6.39	6.64	5.27	7.60	
			Total	325	6.593	0.654	6.52	6.66	4.47	10.13	
		3	No	239	8.257	0.660	8.17	8.34	6.24	9.87	0.670
			Yes	87	8.221	0.680	8.08	8.37	6.18	9.55	
			Total	326	8.248	0.664	8.18	8.32	6.18	9.87	
		4	No	239	9.349	0.617	9.27	9.43	6.41	10.74	0.805
			Yes	84	9.330	0.592	9.20	9.46	8.01	10.44	
			Total	323	9.344	0.610	9.28	9.41	6.41	10.74	
		5	No	241	9.454	0.636	9.37	9.53	6.01	10.99	0.512
			Yes	88	9.506	0.627	9.37	9.64	7.90	10.91	
			Total	329	9.468	0.633	9.40	9.54	6.01	10.99	
		6	No	241	11.403	0.611	11.33	11.48	9.73	13.17	0.999
			Yes	89	11.403	0.632	11.27	11.54	9.81	12.76	
			Total	330	11.403	0.616	11.34	11.47	9.73	13.17	
		7	No	236	11.361	0.783	11.26	11.46	8.98	13.32	0.918
			Yes	88	11.351	0.737	11.19	11.51	9.34	13.04	
			Total	324	11.358	0.770	11.27	11.44	8.98	13.32	
	Mesiodistal	1	No	242	8.687	0.602	8.61	8.76	6.64	10.47	0.771
			Yes	89	8.667	0.466	8.57	8.76	7.47	10.13	
			Total	331	8.682	0.568	8.62	8.74	6.64	10.47	
		2	No	237	6.781	0.653	6.70	6.86	3.90	8.07	0.726
			Yes	88	6.753	0.602	6.63	6.88	4.75	8.10	
			Total	325	6.773	0.639	6.70	6.84	3.90	8.10	
		3	No	239	7.701	0.446	7.64	7.76	6.78	9.14	0.375
			Yes	89	7.649	0.521	7.54	7.76	5.57	8.84	
			Total	328	7.687	0.467	7.64	7.74	5.57	9.14	
		4	No	239	6.977	0.480	6.92	7.04	4.94	9.03	0.870
			Yes	84	6.967	0.497	6.86	7.07	5.58	8.08	
			Total	323	6.974	0.484	6.92	7.03	4.94	9.03	
		5	No	241	6.670	0.507	6.61	6.73	4.97	8.96	0.660
			Yes	88	6.698	0.514	6.59	6.81	5.19	8.57	
			Total	329	6.677	0.508	6.62	6.73	4.97	8.96	
		6	No	241	10.090	0.607	10.01	10.17	8.46	11.70	0.424
			Yes	89	10.152	0.694	10.01	10.30	8.47	11.89	
			Total	330	10.106	0.631	10.04	10.17	8.46	11.89	
		7	No	236	9.857	0.595	9.78	9.93	8.26	11.59	0.405
			Yes	88	9.921	0.668	9.78	10.06	8.23	11.62	
			Total	324	9.874	0.616	9.81	9.94	8.23	11.62	

Mandible	Buccolingual	1	No	241	6.279	0.507	6.21	6.34	4.78	7.82	0.295
			Yes	88	6.216	0.401	6.13	6.30	5.19	7.04	
			Total	329	6.262	0.481	6.21	6.31	4.78	7.82	
		2	No	242	6.559	0.512	6.49	6.62	5.17	7.97	0.052
			Yes	89	6.440	0.439	6.35	6.53	5.46	7.33	
			Total	331	6.527	0.496	6.47	6.58	5.17	7.97	
		3	No	242	7.499	0.632	7.42	7.58	5.89	9.43	0.754
			Yes	89	7.475	0.605	7.35	7.60	6.07	8.65	
			Total	331	7.492	0.624	7.42	7.56	5.89	9.43	
		4	No	241	8.025	0.564	7.95	8.10	6.11	9.32	0.696
			Yes	86	8.054	0.635	7.92	8.19	6.63	9.71	
			Total	327	8.033	0.583	7.97	8.10	6.11	9.71	
		5	No	239	8.730	0.577	8.66	8.80	6.56	9.95	0.955
			Yes	87	8.726	0.574	8.60	8.85	7.33	10.18	
			Total	326	8.729	0.575	8.67	8.79	6.56	10.18	
		6	No	242	10.767	0.551	10.70	10.84	8.82	12.62	0.220
			Yes	88	10.683	0.539	10.57	10.80	9.32	11.88	
			Total	330	10.745	0.548	10.69	10.80	8.82	12.62	
		7	No	238	10.565	0.625	10.48	10.64	8.56	12.04	0.824
			Yes	89	10.548	0.578	10.43	10.67	9.26	11.75	
			Total	327	10.560	0.612	10.49	10.63	8.56	12.04	
	Mesiodistal	1	No	242	5.412	0.405	5.36	5.46	4.12	6.45	0.510
			Yes	89	5.379	0.393	5.30	5.46	4.47	6.27	
			Total	331	5.403	0.402	5.36	5.45	4.12	6.45	
		2	No	242	5.969	0.414	5.92	6.02	4.50	7.22	0.387
			Yes	89	5.924	0.430	5.83	6.01	4.75	6.98	
			Total	331	5.957	0.418	5.91	6.00	4.50	7.22	
		3	No	242	6.695	0.442	6.64	6.75	5.39	7.98	0.785
			Yes	89	6.680	0.464	6.58	6.78	5.45	8.00	
			Total	331	6.691	0.447	6.64	6.74	5.39	8.00	
		4	No	241	7.067	0.462	7.01	7.13	5.44	8.30	0.703
			Yes	86	7.044	0.526	6.93	7.16	5.67	8.26	
			Total	327	7.061	0.479	7.01	7.11	5.44	8.30	
		5	No	239	7.121	0.522	7.05	7.19	5.86	8.79	0.984
			Yes	88	7.120	0.523	7.01	7.23	6.09	8.62	
			Total	327	7.121	0.521	7.06	7.18	5.86	8.79	
		6	No	242	10.848	0.673	10.76	10.93	8.78	12.99	0.515
			Yes	88	10.792	0.733	10.64	10.95	8.17	12.65	
			Total	330	10.833	0.689	10.76	10.91	8.17	12.99	
		7	No	238	10.278	0.640	10.20	10.36	8.52	12.31	0.397
			Yes	89	10.345	0.625	10.21	10.48	8.95	11.84	
			Total	327	10.297	0.635	10.23	10.37	8.52	12.31	

Tooth numbers 1 to 7 indicate the central to the second molar teeth. SD: standard deviation; CI: confidence interval; Min: minimum; Max: maximum.

**Table 6:** Descriptive statistics and 95% CIs for dental sizes (averages of the right and left sides, mm) in cases with and without microdontia. The groups are compared using the *t*-test.

Jaw	Measurement	Tooth	Microdontia	*N*	Mean	SD	95% CI		Min	Max	*P*
Maxilla	Buccolingual	1	No	203	7.489	0.550	7.41	7.56	5.91	9.04	<0.0005
			Yes	128	7.250	0.584	7.15	7.35	5.88	8.52	
			Total	331	7.396	0.575	7.33	7.46	5.88	9.04	
		2	No	199	6.681	0.650	6.59	6.77	4.51	10.13	0.002
			Yes	126	6.454	0.639	6.34	6.57	4.47	8.19	
			Total	325	6.593	0.654	6.52	6.66	4.47	10.13	
		3	No	200	8.370	0.659	8.28	8.46	6.18	9.87	<0.0005
			Yes	126	8.054	0.628	7.94	8.16	6.24	9.54	
			Total	326	8.248	0.664	8.18	8.32	6.18	9.87	
		4	No	196	9.483	0.525	9.41	9.56	8.01	10.74	<0.0005
			Yes	127	9.129	0.668	9.01	9.25	6.41	10.68	
			Total	323	9.344	0.610	9.28	9.41	6.41	10.74	
		5	No	202	9.663	0.508	9.59	9.73	8.36	10.99	<0.0005
			Yes	127	9.156	0.689	9.04	9.28	6.01	10.78	
			Total	329	9.468	0.633	9.40	9.54	6.01	10.99	
		6	No	202	11.584	0.558	11.51	11.66	10.11	13.17	<0.0005
			Yes	128	11.117	0.595	11.01	11.22	9.73	12.47	
			Total	330	11.403	0.616	11.34	11.47	9.73	13.17	
		7	No	197	11.609	0.678	11.51	11.70	9.54	13.32	<0.0005
			Yes	127	10.969	0.743	10.84	11.10	8.98	12.89	
			Total	324	11.358	0.770	11.27	11.44	8.98	13.32	
	Mesiodistal	1	No	203	8.834	0.514	8.76	8.91	7.63	10.47	<0.0005
			Yes	128	8.440	0.567	8.34	8.54	6.64	10.33	
			Total	331	8.682	0.568	8.62	8.74	6.64	10.47	
		2	No	199	6.977	0.513	6.91	7.05	4.74	8.10	<0.0005
			Yes	126	6.451	0.685	6.33	6.57	3.90	7.70	
			Total	325	6.773	0.639	6.70	6.84	3.90	8.10	
		3	No	201	7.826	0.444	7.76	7.89	6.84	9.14	<0.0005
			Yes	127	7.467	0.416	7.39	7.54	5.57	8.47	
			Total	328	7.687	0.467	7.64	7.74	5.57	9.14	
		4	No	196	7.107	0.430	7.05	7.17	5.58	8.29	<0.0005
			Yes	127	6.770	0.492	6.68	6.86	4.94	9.03	
			Total	323	6.974	0.484	6.92	7.03	4.94	9.03	
		5	No	202	6.817	0.469	6.75	6.88	5.70	8.57	<0.0005
			Yes	127	6.455	0.490	6.37	6.54	4.97	8.96	
			Total	329	6.677	0.508	6.62	6.73	4.97	8.96	
		6	No	202	10.318	0.588	10.24	10.40	8.74	11.89	<0.0005
			Yes	128	9.773	0.549	9.68	9.87	8.46	11.12	
			Total	330	10.106	0.631	10.04	10.17	8.46	11.89	
		7	No	197	10.100	0.568	10.02	10.18	8.59	11.62	<0.0005
			Yes	127	9.524	0.517	9.43	9.61	8.23	11.05	
			Total	324	9.874	0.616	9.81	9.94	8.23	11.62	

Mandible	Buccolingual	1	No	201	6.329	0.470	6.26	6.39	4.78	7.82	0.001
			Yes	128	6.157	0.481	6.07	6.24	4.83	7.37	
			Total	329	6.262	0.481	6.21	6.31	4.78	7.82	
		2	No	203	6.598	0.472	6.53	6.66	5.21	7.97	0.001
			Yes	128	6.414	0.513	6.32	6.50	5.17	7.53	
			Total	331	6.527	0.496	6.47	6.58	5.17	7.97	
		3	No	203	7.595	0.650	7.51	7.69	5.89	9.43	<0.0005
			Yes	128	7.329	0.543	7.23	7.42	5.91	8.71	
			Total	331	7.492	0.624	7.42	7.56	5.89	9.43	
		4	No	200	8.169	0.535	8.09	8.24	6.97	9.59	<0.0005
			Yes	127	7.818	0.592	7.71	7.92	6.11	9.71	
			Total	327	8.033	0.583	7.97	8.10	6.11	9.71	
		5	No	198	8.891	0.510	8.82	8.96	7.33	9.95	<0.0005
			Yes	128	8.479	0.581	8.38	8.58	6.56	10.18	
			Total	326	8.729	0.575	8.67	8.79	6.56	10.18	
		6	No	202	10.914	0.501	10.84	10.98	9.82	12.62	<0.0005
			Yes	128	10.477	0.515	10.39	10.57	8.82	11.60	
			Total	330	10.745	0.548	10.69	10.80	8.82	12.62	
		7	No	199	10.732	0.541	10.66	10.81	9.31	12.04	<0.0005
			Yes	128	10.292	0.621	10.18	10.40	8.56	11.88	
			Total	327	10.560	0.612	10.49	10.63	8.56	12.04	
	Mesiodistal	1	No	203	5.495	0.377	5.44	5.55	4.51	6.45	<0.0005
			Yes	128	5.257	0.397	5.19	5.33	4.12	6.41	
			Total	331	5.403	0.402	5.36	5.45	4.12	6.45	
		2	No	203	6.054	0.387	6.00	6.11	5.18	7.11	<0.0005
			Yes	128	5.803	0.421	5.73	5.88	4.50	7.22	
			Total	331	5.957	0.418	5.91	6.00	4.50	7.22	
		3	No	203	6.811	0.424	6.75	6.87	5.74	7.98	<0.0005
			Yes	128	6.502	0.418	6.43	6.58	5.39	8.00	
			Total	331	6.691	0.447	6.64	6.74	5.39	8.00	
		4	No	200	7.207	0.430	7.15	7.27	6.06	8.30	<0.0005
			Yes	127	6.831	0.462	6.75	6.91	5.44	7.80	
			Total	327	7.061	0.479	7.01	7.11	5.44	8.30	
		5	No	199	7.286	0.500	7.22	7.36	6.19	8.79	<0.0005
			Yes	128	6.864	0.445	6.79	6.94	5.86	8.13	
			Total	327	7.121	0.521	7.06	7.18	5.86	8.79	
		6	No	202	11.066	0.606	10.98	11.15	9.41	12.99	<0.0005
			Yes	128	10.467	0.652	10.35	10.58	8.17	12.15	
			Total	330	10.833	0.689	10.76	10.91	8.17	12.99	
		7	No	199	10.508	0.567	10.43	10.59	9.18	12.31	<0.0005
			Yes	128	9.968	0.597	9.86	10.07	8.52	11.84	
			Total	327	10.297	0.635	10.23	10.37	8.52	12.31	

Tooth numbers 1 to 7 denote the central to the second molar teeth. SD: standard deviation; CI: confidence interval; Min: minimum; Max: maximum.

**Table 7:** The Bolton ratios in men, women, and different Angle classes.

Bolton ratio	Variables	*N*	Mean	SD	95% CI		Min	Max	*P*
Overall	Female	210	91.78	2.48	91.44	92.12	83.97	99.09	0.229
	Male	58	92.22	2.42	91.58	92.85	86.32	99.87	
	Total	268	91.87	2.47	91.58	92.17	83.97	99.87	

Anterior	Female	210	77.86	3.11	77.44	78.29	69.00	89.43	0.059
	Male	58	78.74	3.02	77.94	79.53	71.45	87.61	
	Total	268	78.05	3.11	77.68	78.43	69.00	89.43	

Posterior	Female	210	105.42	3.77	104.91	105.93	96.20	114.43	0.995
	Male	58	105.41	3.83	104.41	106.42	97.14	115.59	
	Total	268	105.42	3.77	104.96	105.87	96.20	115.59	

Overall	Class I	142	91.96	2.43	91.56	92.37	86.32	99.09	0.083
	Class II	110	91.55	2.41	91.10	92.01	83.97	99.87	
	Class III	13	93.03	2.50	91.52	94.54	88.94	97.73	

Anterior	Class I	142	77.90	2.98	77.41	78.40	71.45	86.06	0.667
	Class II	110	78.16	3.30	77.54	78.79	69.00	89.43	
	Class III	13	78.56	2.96	76.77	80.35	74.48	85.40	

Posterior	Class I	142	105.80	3.64	105.19	106.40	96.78	112.49	0.008
	Class II	110	104.60	3.69	103.90	105.30	96.20	115.59	
	Class III	13	107.19	4.16	104.67	109.70	100.42	112.31	

SD: standard deviation; CI: confidence interval; Min: minimum; Max: maximum. The *P* values for comparisons between men and women are calculated using the unpaired *t*-test. The *P* values for comparisons across Angle classes are calculated using the one-way ANOVA.

**Table 8:** The Bolton indices in men versus women within different Angle classes.

Angle classes	Bolton ratio	Sex	*N*	Mean	SD	95% CI		Min	Max	*P*
Class I	Overall	Female	115	91.91	2.48	91.45	92.37	86.40	99.09	0.586
		Male	27	92.19	2.22	91.32	93.07	86.32	95.72	
	Anterior	Female	115	77.89	2.97	77.34	78.44	72.16	86.06	0.909
		Male	27	77.96	3.09	76.74	79.18	71.45	86.02	
	Posterior	Female	115	105.69	3.72	105.00	106.38	96.78	112.37	0.472
		Male	27	106.25	3.28	104.96	107.55	99.47	112.49	

Class II	Overall	Female	82	91.35	2.35	90.83	91.86	83.97	97.67	0.121
		Male	28	92.17	2.53	91.19	93.15	87.72	99.87	
	Anterior	Female	82	77.72	3.33	76.99	78.45	69.00	89.43	0.014
		Male	28	79.48	2.92	78.35	80.61	74.52	87.61	
	Posterior	Female	82	104.64	3.60	103.85	105.43	96.20	114.43	0.841
		Male	28	104.48	4.01	102.92	106.03	97.14	115.59	

Class III	Overall	Female	11	93.46	2.48	91.79	95.13	88.94	97.73	—
		Male	2	90.69	0.57	—	—	90.28	91.09	
	Anterior	Female	11	78.72	3.15	76.60	80.83	74.48	85.40	—
		Male	2	77.68	2.09	—	—	76.20	79.15	
	Posterior	Female	11	107.94	4.09	105.20	110.69	100.42	112.31	—
		Male	2	103.03	0.46	—	—	102.71	103.36	

SD: standard deviation; CI: confidence interval; Min: minimum; Max: maximum. The *P* values are calculated using the unpaired *t*-test.

## Data Availability

The raw data are available from the authors upon reasonable request.

## Supplementary Materials

Supplementary Materials Supplementary file: Appendix 1. Online supplementary spreadsheet showing partial correlation coefficients (controlling for the role of sex) across tooth sizes and between age and tooth sizes.
