# Supplementary material for: Buccolingual and Mesiodistal Dimensions of the Permanent Teeth, Their Diagnostic Value for Sex Identification, and Bolton Indices
Source: Biomed Res Int. 2022 Feb 10;2022:8381436. doi: 10.1155/2022/8381436 (PMC8853791; doi:10.1155/2022/8381436)
Supplement: Supplementary Materials — Supplementary file: Appendix 1. Online supplementary spreadsheet showing partial correlation coefficients (controlling for the role of sex) across tooth sizes and between age and tooth sizes. [file 8381436.f1.docx]

**Appendix 1.** Online supplementary spreadsheet showing partial correlation coefficients (controlling for the role of sex) across tooth sizes, and between age and tooth sizes.

| **Dimension** | **Jaw** | **Side** | **Variable** |  | **Age** | **Central** | **Lateral** | **Canine** | **First Premolar** | **Second Premolar** | **First Molar** | **Second Molar** |
| --- | --- | --- | --- | --- | --- | --- | --- | --- | --- | --- | --- | --- |
| **Buccolingual** | **Maxilla** | **Right** | **Age** | **Correlation** |  | 0.055 | 0.033 | 0.043 | -0.119 | -0.121 | -0.073 | 0.045 |
|  |  |  |  | ***P*** |  | 0.353 | 0.581 | 0.472 | 0.045 | 0.040 | 0.218 | 0.443 |
|  |  |  |  | **df** |  | 285 | 285 | 285 | 285 | 285 | 285 | 285 |
|  |  |  | **Central** | **Correlation** | 0.055 |  | 0.583 | 0.534 | 0.462 | 0.405 | 0.426 | 0.414 |
|  |  |  |  | ***P*** | 0.353 |  | 0.000 | 0.000 | 0.000 | 0.000 | 0.000 | 0.000 |
|  |  |  |  | **df** | 285 |  | 285 | 285 | 285 | 285 | 285 | 285 |
|  |  |  | **Lateral** | **Correlation** | 0.033 | 0.583 |  | 0.517 | 0.323 | 0.287 | 0.289 | 0.315 |
|  |  |  |  | ***P*** | 0.581 | 0.000 |  | 0.000 | 0.000 | 0.000 | 0.000 | 0.000 |
|  |  |  |  | **df** | 285 | 285 |  | 285 | 285 | 285 | 285 | 285 |
|  |  |  | **Canine** | **Correlation** | 0.043 | 0.534 | 0.517 |  | 0.503 | 0.509 | 0.507 | 0.550 |
|  |  |  |  | ***P*** | 0.472 | 0.000 | 0.000 |  | 0.000 | 0.000 | 0.000 | 0.000 |
|  |  |  |  | **df** | 285 | 285 | 285 |  | 285 | 285 | 285 | 285 |
|  |  |  | **First Premolar** | **Correlation** | -0.119 | 0.462 | 0.323 | 0.503 |  | 0.757 | 0.576 | 0.510 |
|  |  |  |  | ***P*** | 0.045 | 0.000 | 0.000 | 0.000 |  | 0.000 | 0.000 | 0.000 |
|  |  |  |  | **df** | 285 | 285 | 285 | 285 |  | 285 | 285 | 285 |
|  |  |  | **Second Premolar** | **Correlation** | -0.121 | 0.405 | 0.287 | 0.509 | 0.757 |  | 0.659 | 0.602 |
|  |  |  |  | ***P*** | 0.040 | 0.000 | 0.000 | 0.000 | 0.000 |  | 0.000 | 0.000 |
|  |  |  |  | **df** | 285 | 285 | 285 | 285 | 285 |  | 285 | 285 |
|  |  |  | **First Molar** | **Correlation** | -0.073 | 0.426 | 0.289 | 0.507 | 0.576 | 0.659 |  | 0.759 |
|  |  |  |  | ***P*** | 0.218 | 0.000 | 0.000 | 0.000 | 0.000 | 0.000 |  | 0.000 |
|  |  |  |  | **df** | 285 | 285 | 285 | 285 | 285 | 285 |  | 285 |
|  |  |  | **Second Molar** | **Correlation** | 0.045 | 0.414 | 0.315 | 0.550 | 0.510 | 0.602 | 0.759 |  |
|  |  |  |  | ***P*** | 0.443 | 0.000 | 0.000 | 0.000 | 0.000 | 0.000 | 0.000 |  |
|  |  |  |  | **df** | 285 | 285 | 285 | 285 | 285 | 285 | 285 |  |
|  |  | **Left** | **Age** | **Correlation** |  | 0.027 | -0.041 | 0.027 | -0.131 | -0.145 | -0.068 | 0.063 |
|  |  |  |  | ***P*** |  | 0.644 | 0.481 | 0.642 | 0.025 | 0.013 | 0.249 | 0.282 |
|  |  |  |  | **df** |  | 290 | 290 | 290 | 290 | 290 | 290 | 290 |
|  |  |  | **Central** | **Correlation** | 0.027 |  | 0.561 | 0.506 | 0.394 | 0.265 | 0.434 | 0.397 |
|  |  |  |  | ***P*** | 0.644 |  | 0.000 | 0.000 | 0.000 | 0.000 | 0.000 | 0.000 |
|  |  |  |  | **df** | 290 |  | 290 | 290 | 290 | 290 | 290 | 290 |
|  |  |  | **Lateral** | **Correlation** | -0.041 | 0.561 |  | 0.475 | 0.349 | 0.216 | 0.361 | 0.306 |
|  |  |  |  | ***P*** | 0.481 | 0.000 |  | 0.000 | 0.000 | 0.000 | 0.000 | 0.000 |
|  |  |  |  | **df** | 290 | 290 |  | 290 | 290 | 290 | 290 | 290 |
|  |  |  | **Canine** | **Correlation** | 0.027 | 0.506 | 0.475 |  | 0.506 | 0.448 | 0.535 | 0.542 |
|  |  |  |  | ***P*** | 0.642 | 0.000 | 0.000 |  | 0.000 | 0.000 | 0.000 | 0.000 |
|  |  |  |  | **df** | 290 | 290 | 290 |  | 290 | 290 | 290 | 290 |
|  |  |  | **First Premolar** | **Correlation** | -0.131 | 0.394 | 0.349 | 0.506 |  | 0.797 | 0.621 | 0.504 |
|  |  |  |  | ***P*** | 0.025 | 0.000 | 0.000 | 0.000 |  | 0.000 | 0.000 | 0.000 |
|  |  |  |  | **df** | 290 | 290 | 290 | 290 |  | 290 | 290 | 290 |
|  |  |  | **Second Premolar** | **Correlation** | -0.145 | 0.265 | 0.216 | 0.448 | 0.797 |  | 0.648 | 0.552 |
|  |  |  |  | ***P*** | 0.013 | 0.000 | 0.000 | 0.000 | 0.000 |  | 0.000 | 0.000 |
|  |  |  |  | **df** | 290 | 290 | 290 | 290 | 290 |  | 290 | 290 |
|  |  |  | **First Molar** | **Correlation** | -0.068 | 0.434 | 0.361 | 0.535 | 0.621 | 0.648 |  | 0.738 |
|  |  |  |  | ***P*** | 0.249 | 0.000 | 0.000 | 0.000 | 0.000 | 0.000 |  | 0.000 |
|  |  |  |  | **df** | 290 | 290 | 290 | 290 | 290 | 290 |  | 290 |
|  |  |  | **Second Molar** | **Correlation** | 0.063 | 0.397 | 0.306 | 0.542 | 0.504 | 0.552 | 0.738 |  |
|  |  |  |  | ***P*** | 0.282 | 0.000 | 0.000 | 0.000 | 0.000 | 0.000 | 0.000 |  |
|  |  |  |  | **df** | 290 | 290 | 290 | 290 | 290 | 290 | 290 |  |
|  | **Mandible** | **Right** | **Age** | **Correlation** |  | 0.006 | 0.076 | 0.049 | -0.093 | -0.138 | -0.155 | 0.017 |
|  |  |  |  | ***P*** |  | 0.920 | 0.192 | 0.401 | 0.109 | 0.017 | 0.007 | 0.771 |
|  |  |  |  | **df** |  | 295 | 295 | 295 | 295 | 295 | 295 | 295 |
|  |  |  | **Central** | **Correlation** | 0.006 |  | 0.705 | 0.500 | 0.422 | 0.406 | 0.375 | 0.391 |
|  |  |  |  | ***P*** | 0.920 |  | 0.000 | 0.000 | 0.000 | 0.000 | 0.000 | 0.000 |
|  |  |  |  | **df** | 295 |  | 295 | 295 | 295 | 295 | 295 | 295 |
|  |  |  | **Lateral** | **Correlation** | 0.076 | 0.705 |  | 0.577 | 0.460 | 0.450 | 0.428 | 0.447 |
|  |  |  |  | ***P*** | 0.192 | 0.000 |  | 0.000 | 0.000 | 0.000 | 0.000 | 0.000 |
|  |  |  |  | **df** | 295 | 295 |  | 295 | 295 | 295 | 295 | 295 |
|  |  |  | **Canine** | **Correlation** | 0.049 | 0.500 | 0.577 |  | 0.494 | 0.431 | 0.403 | 0.418 |
|  |  |  |  | ***P*** | 0.401 | 0.000 | 0.000 |  | 0.000 | 0.000 | 0.000 | 0.000 |
|  |  |  |  | **df** | 295 | 295 | 295 |  | 295 | 295 | 295 | 295 |
|  |  |  | **First Premolar** | **Correlation** | -0.093 | 0.422 | 0.460 | 0.494 |  | 0.721 | 0.524 | 0.512 |
|  |  |  |  | ***P*** | 0.109 | 0.000 | 0.000 | 0.000 |  | 0.000 | 0.000 | 0.000 |
|  |  |  |  | **df** | 295 | 295 | 295 | 295 |  | 295 | 295 | 295 |
|  |  |  | **Second Premolar** | **Correlation** | -0.138 | 0.406 | 0.450 | 0.431 | 0.721 |  | 0.584 | 0.544 |
|  |  |  |  | ***P*** | 0.017 | 0.000 | 0.000 | 0.000 | 0.000 |  | 0.000 | 0.000 |
|  |  |  |  | **df** | 295 | 295 | 295 | 295 | 295 |  | 295 | 295 |
|  |  |  | **First Molar** | **Correlation** | -0.155 | 0.375 | 0.428 | 0.403 | 0.524 | 0.584 |  | 0.703 |
|  |  |  |  | ***P*** | 0.007 | 0.000 | 0.000 | 0.000 | 0.000 | 0.000 |  | 0.000 |
|  |  |  |  | **df** | 295 | 295 | 295 | 295 | 295 | 295 |  | 295 |
|  |  |  | **Second Molar** | **Correlation** | 0.017 | 0.391 | 0.447 | 0.418 | 0.512 | 0.544 | 0.703 |  |
|  |  |  |  | ***P*** | 0.771 | 0.000 | 0.000 | 0.000 | 0.000 | 0.000 | 0.000 |  |
|  |  |  |  | **df** | 295 | 295 | 295 | 295 | 295 | 295 | 295 |  |
|  |  | **Left** | **Age** | **Correlation** |  | 0.023 | 0.083 | 0.093 | -0.112 | -0.131 | -0.135 | 0.031 |
|  |  |  |  | ***P*** |  | 0.689 | 0.152 | 0.106 | 0.053 | 0.023 | 0.019 | 0.595 |
|  |  |  |  | **df** |  | 300 | 300 | 300 | 300 | 300 | 300 | 300 |
|  |  |  | **Central** | **Correlation** | 0.023 |  | 0.698 | 0.489 | 0.398 | 0.421 | 0.408 | 0.364 |
|  |  |  |  | ***P*** | 0.689 |  | 0.000 | 0.000 | 0.000 | 0.000 | 0.000 | 0.000 |
|  |  |  |  | **df** | 300 |  | 300 | 300 | 300 | 300 | 300 | 300 |
|  |  |  | **Lateral** | **Correlation** | 0.083 | 0.698 |  | 0.578 | 0.452 | 0.413 | 0.378 | 0.352 |
|  |  |  |  | ***P*** | 0.152 | 0.000 |  | 0.000 | 0.000 | 0.000 | 0.000 | 0.000 |
|  |  |  |  | **df** | 300 | 300 |  | 300 | 300 | 300 | 300 | 300 |
|  |  |  | **Canine** | **Correlation** | 0.093 | 0.489 | 0.578 |  | 0.510 | 0.475 | 0.380 | 0.471 |
|  |  |  |  | ***P*** | 0.106 | 0.000 | 0.000 |  | 0.000 | 0.000 | 0.000 | 0.000 |
|  |  |  |  | **df** | 300 | 300 | 300 |  | 300 | 300 | 300 | 300 |
|  |  |  | **First Premolar** | **Correlation** | -0.112 | 0.398 | 0.452 | 0.510 |  | 0.680 | 0.594 | 0.554 |
|  |  |  |  | ***P*** | 0.053 | 0.000 | 0.000 | 0.000 |  | 0.000 | 0.000 | 0.000 |
|  |  |  |  | **df** | 300 | 300 | 300 | 300 |  | 300 | 300 | 300 |
|  |  |  | **Second Premolar** | **Correlation** | -0.131 | 0.421 | 0.413 | 0.475 | 0.680 |  | 0.602 | 0.542 |
|  |  |  |  | ***P*** | 0.023 | 0.000 | 0.000 | 0.000 | 0.000 |  | 0.000 | 0.000 |
|  |  |  |  | **df** | 300 | 300 | 300 | 300 | 300 |  | 300 | 300 |
|  |  |  | **First Molar** | **Correlation** | -0.135 | 0.408 | 0.378 | 0.380 | 0.594 | 0.602 |  | 0.728 |
|  |  |  |  | ***P*** | 0.019 | 0.000 | 0.000 | 0.000 | 0.000 | 0.000 |  | 0.000 |
|  |  |  |  | **df** | 300 | 300 | 300 | 300 | 300 | 300 |  | 300 |
|  |  |  | **Second Molar** | **Correlation** | 0.031 | 0.364 | 0.352 | 0.471 | 0.554 | 0.542 | 0.728 |  |
|  |  |  |  | ***P*** | 0.595 | 0.000 | 0.000 | 0.000 | 0.000 | 0.000 | 0.000 |  |
|  |  |  |  | **df** | 300 | 300 | 300 | 300 | 300 | 300 | 300 |  |
| **Mesiodistal** | **Maxilla** | **Right** | **Age** | **Correlation** |  | -0.132 | -0.035 | -0.097 | -0.124 | -0.084 | -0.185 | -0.111 |
|  |  |  |  | ***P*** |  | 0.025 | 0.554 | 0.100 | 0.034 | 0.156 | 0.002 | 0.060 |
|  |  |  |  | **df** |  | 287 | 287 | 287 | 287 | 287 | 287 | 287 |
|  |  |  | **Central** | **Correlation** | -0.132 |  | 0.514 | 0.441 | 0.333 | 0.362 | 0.336 | 0.421 |
|  |  |  |  | ***P*** | 0.025 |  | 0.000 | 0.000 | 0.000 | 0.000 | 0.000 | 0.000 |
|  |  |  |  | **df** | 287 |  | 287 | 287 | 287 | 287 | 287 | 287 |
|  |  |  | **Lateral** | **Correlation** | -0.035 | 0.514 |  | 0.353 | 0.294 | 0.322 | 0.167 | 0.225 |
|  |  |  |  | ***P*** | 0.554 | 0.000 |  | 0.000 | 0.000 | 0.000 | 0.004 | 0.000 |
|  |  |  |  | **df** | 287 | 287 |  | 287 | 287 | 287 | 287 | 287 |
|  |  |  | **Canine** | **Correlation** | -0.097 | 0.441 | 0.353 |  | 0.527 | 0.463 | 0.283 | 0.257 |
|  |  |  |  | ***P*** | 0.100 | 0.000 | 0.000 |  | 0.000 | 0.000 | 0.000 | 0.000 |
|  |  |  |  | **df** | 287 | 287 | 287 |  | 287 | 287 | 287 | 287 |
|  |  |  | **First Premolar** | **Correlation** | -0.124 | 0.333 | 0.294 | 0.527 |  | 0.694 | 0.340 | 0.418 |
|  |  |  |  | ***P*** | 0.034 | 0.000 | 0.000 | 0.000 |  | 0.000 | 0.000 | 0.000 |
|  |  |  |  | **df** | 287 | 287 | 287 | 287 |  | 287 | 287 | 287 |
|  |  |  | **Second Premolar** | **Correlation** | -0.084 | 0.362 | 0.322 | 0.463 | 0.694 |  | 0.392 | 0.454 |
|  |  |  |  | ***P*** | 0.156 | 0.000 | 0.000 | 0.000 | 0.000 |  | 0.000 | 0.000 |
|  |  |  |  | **df** | 287 | 287 | 287 | 287 | 287 |  | 287 | 287 |
|  |  |  | **First Molar** | **Correlation** | -0.185 | 0.336 | 0.167 | 0.283 | 0.340 | 0.392 |  | 0.609 |
|  |  |  |  | ***P*** | 0.002 | 0.000 | 0.004 | 0.000 | 0.000 | 0.000 |  | 0.000 |
|  |  |  |  | **df** | 287 | 287 | 287 | 287 | 287 | 287 |  | 287 |
|  |  |  | **Second Molar** | **Correlation** | -0.111 | 0.421 | 0.225 | 0.257 | 0.418 | 0.454 | 0.609 |  |
|  |  |  |  | ***P*** | 0.060 | 0.000 | 0.000 | 0.000 | 0.000 | 0.000 | 0.000 |  |
|  |  |  |  | **df** | 287 | 287 | 287 | 287 | 287 | 287 | 287 |  |
|  |  | **Left** | **Age** | **Correlation** |  | -0.159 | -0.095 | -0.129 | -0.133 | -0.083 | -0.134 | -0.062 |
|  |  |  |  | ***P*** |  | 0.006 | 0.106 | 0.027 | 0.023 | 0.154 | 0.022 | 0.293 |
|  |  |  |  | **df** |  | 291 | 291 | 291 | 291 | 291 | 291 | 291 |
|  |  |  | **Central** | **Correlation** | -0.159 |  | 0.542 | 0.359 | 0.338 | 0.221 | 0.257 | 0.241 |
|  |  |  |  | ***P*** | 0.006 |  | 0.000 | 0.000 | 0.000 | 0.000 | 0.000 | 0.000 |
|  |  |  |  | **df** | 291 |  | 291 | 291 | 291 | 291 | 291 | 291 |
|  |  |  | **Lateral** | **Correlation** | -0.095 | 0.542 |  | 0.420 | 0.471 | 0.346 | 0.204 | 0.221 |
|  |  |  |  | ***P*** | 0.106 | 0.000 |  | 0.000 | 0.000 | 0.000 | 0.000 | 0.000 |
|  |  |  |  | **df** | 291 | 291 |  | 291 | 291 | 291 | 291 | 291 |
|  |  |  | **Canine** | **Correlation** | -0.129 | 0.359 | 0.420 |  | 0.516 | 0.374 | 0.293 | 0.347 |
|  |  |  |  | ***P*** | 0.027 | 0.000 | 0.000 |  | 0.000 | 0.000 | 0.000 | 0.000 |
|  |  |  |  | **df** | 291 | 291 | 291 |  | 291 | 291 | 291 | 291 |
|  |  |  | **First Premolar** | **Correlation** | -0.133 | 0.338 | 0.471 | 0.516 |  | 0.680 | 0.392 | 0.393 |
|  |  |  |  | ***P*** | 0.023 | 0.000 | 0.000 | 0.000 |  | 0.000 | 0.000 | 0.000 |
|  |  |  |  | **df** | 291 | 291 | 291 | 291 |  | 291 | 291 | 291 |
|  |  |  | **Second Premolar** | **Correlation** | -0.083 | 0.221 | 0.346 | 0.374 | 0.680 |  | 0.354 | 0.422 |
|  |  |  |  | ***P*** | 0.154 | 0.000 | 0.000 | 0.000 | 0.000 |  | 0.000 | 0.000 |
|  |  |  |  | **df** | 291 | 291 | 291 | 291 | 291 |  | 291 | 291 |
|  |  |  | **First Molar** | **Correlation** | -0.134 | 0.257 | 0.204 | 0.293 | 0.392 | 0.354 |  | 0.589 |
|  |  |  |  | ***P*** | 0.022 | 0.000 | 0.000 | 0.000 | 0.000 | 0.000 |  | 0.000 |
|  |  |  |  | **df** | 291 | 291 | 291 | 291 | 291 | 291 |  | 291 |
|  |  |  | **Second Molar** | **Correlation** | -0.062 | 0.241 | 0.221 | 0.347 | 0.393 | 0.422 | 0.589 |  |
|  |  |  |  | ***P*** | 0.293 | 0.000 | 0.000 | 0.000 | 0.000 | 0.000 | 0.000 |  |
|  |  |  |  | **df** | 291 | 291 | 291 | 291 | 291 | 291 | 291 |  |
|  | **Mandible** | **Right** | **Age** | **Correlation** |  | -0.082 | -0.177 | -0.095 | -0.149 | -0.060 | -0.159 | 0.052 |
|  |  |  |  | ***P*** |  | 0.156 | 0.002 | 0.100 | 0.010 | 0.299 | 0.006 | 0.374 |
|  |  |  |  | **df** |  | 298 | 298 | 298 | 298 | 298 | 298 | 298 |
|  |  |  | **Central** | **Correlation** | -0.082 |  | 0.678 | 0.544 | 0.477 | 0.526 | 0.362 | 0.392 |
|  |  |  |  | ***P*** | 0.156 |  | 0.000 | 0.000 | 0.000 | 0.000 | 0.000 | 0.000 |
|  |  |  |  | **df** | 298 |  | 298 | 298 | 298 | 298 | 298 | 298 |
|  |  |  | **Lateral** | **Correlation** | -0.177 | 0.678 |  | 0.614 | 0.531 | 0.500 | 0.431 | 0.455 |
|  |  |  |  | ***P*** | 0.002 | 0.000 |  | 0.000 | 0.000 | 0.000 | 0.000 | 0.000 |
|  |  |  |  | **df** | 298 | 298 |  | 298 | 298 | 298 | 298 | 298 |
|  |  |  | **Canine** | **Correlation** | -0.095 | 0.544 | 0.614 |  | 0.611 | 0.530 | 0.393 | 0.391 |
|  |  |  |  | ***P*** | 0.100 | 0.000 | 0.000 |  | 0.000 | 0.000 | 0.000 | 0.000 |
|  |  |  |  | **df** | 298 | 298 | 298 |  | 298 | 298 | 298 | 298 |
|  |  |  | **First Premolar** | **Correlation** | -0.149 | 0.477 | 0.531 | 0.611 |  | 0.669 | 0.476 | 0.497 |
|  |  |  |  | ***P*** | 0.010 | 0.000 | 0.000 | 0.000 |  | 0.000 | 0.000 | 0.000 |
|  |  |  |  | **df** | 298 | 298 | 298 | 298 |  | 298 | 298 | 298 |
|  |  |  | **Second Premolar** | **Correlation** | -0.060 | 0.526 | 0.500 | 0.530 | 0.669 |  | 0.437 | 0.521 |
|  |  |  |  | ***P*** | 0.299 | 0.000 | 0.000 | 0.000 | 0.000 |  | 0.000 | 0.000 |
|  |  |  |  | **df** | 298 | 298 | 298 | 298 | 298 |  | 298 | 298 |
|  |  |  | **First Molar** | **Correlation** | -0.159 | 0.362 | 0.431 | 0.393 | 0.476 | 0.437 |  | 0.512 |
|  |  |  |  | ***P*** | 0.006 | 0.000 | 0.000 | 0.000 | 0.000 | 0.000 |  | 0.000 |
|  |  |  |  | **df** | 298 | 298 | 298 | 298 | 298 | 298 |  | 298 |
|  |  |  | **Second Molar** | **Correlation** | 0.052 | 0.392 | 0.455 | 0.391 | 0.497 | 0.521 | 0.512 |  |
|  |  |  |  | ***P*** | 0.374 | 0.000 | 0.000 | 0.000 | 0.000 | 0.000 | 0.000 |  |
|  |  |  |  | **df** | 298 | 298 | 298 | 298 | 298 | 298 | 298 |  |
|  |  | **Left** | **Age** | **Correlation** |  | -0.163 | -0.131 | -0.056 | -0.175 | -0.063 | -0.053 | 0.020 |
|  |  |  |  | ***P*** |  | 0.004 | 0.022 | 0.333 | 0.002 | 0.272 | 0.353 | 0.729 |
|  |  |  |  | **df** |  | 304 | 304 | 304 | 304 | 304 | 304 | 304 |
|  |  |  | **Central** | **Correlation** | -0.163 |  | 0.728 | 0.487 | 0.521 | 0.440 | 0.349 | 0.319 |
|  |  |  |  | ***P*** | 0.004 |  | 0.000 | 0.000 | 0.000 | 0.000 | 0.000 | 0.000 |
|  |  |  |  | **df** | 304 |  | 304 | 304 | 304 | 304 | 304 | 304 |
|  |  |  | **Lateral** | **Correlation** | -0.131 | 0.728 |  | 0.485 | 0.505 | 0.431 | 0.345 | 0.289 |
|  |  |  |  | ***P*** | 0.022 | 0.000 |  | 0.000 | 0.000 | 0.000 | 0.000 | 0.000 |
|  |  |  |  | **df** | 304 | 304 |  | 304 | 304 | 304 | 304 | 304 |
|  |  |  | **Canine** | **Correlation** | -0.056 | 0.487 | 0.485 |  | 0.525 | 0.403 | 0.322 | 0.286 |
|  |  |  |  | ***P*** | 0.333 | 0.000 | 0.000 |  | 0.000 | 0.000 | 0.000 | 0.000 |
|  |  |  |  | **df** | 304 | 304 | 304 |  | 304 | 304 | 304 | 304 |
|  |  |  | **First Premolar** | **Correlation** | -0.175 | 0.521 | 0.505 | 0.525 |  | 0.631 | 0.463 | 0.426 |
|  |  |  |  | ***P*** | 0.002 | 0.000 | 0.000 | 0.000 |  | 0.000 | 0.000 | 0.000 |
|  |  |  |  | **df** | 304 | 304 | 304 | 304 |  | 304 | 304 | 304 |
|  |  |  | **Second Premolar** | **Correlation** | -0.063 | 0.440 | 0.431 | 0.403 | 0.631 |  | 0.420 | 0.465 |
|  |  |  |  | ***P*** | 0.272 | 0.000 | 0.000 | 0.000 | 0.000 |  | 0.000 | 0.000 |
|  |  |  |  | **df** | 304 | 304 | 304 | 304 | 304 |  | 304 | 304 |
|  |  |  | **First Molar** | **Correlation** | -0.053 | 0.349 | 0.345 | 0.322 | 0.463 | 0.420 |  | 0.519 |
|  |  |  |  | ***P*** | 0.353 | 0.000 | 0.000 | 0.000 | 0.000 | 0.000 |  | 0.000 |
|  |  |  |  | **df** | 304 | 304 | 304 | 304 | 304 | 304 |  | 304 |
|  |  |  | **Second Molar** | **Correlation** | 0.020 | 0.319 | 0.289 | 0.286 | 0.426 | 0.465 | 0.519 |  |
|  |  |  |  | ***P*** | 0.729 | 0.000 | 0.000 | 0.000 | 0.000 | 0.000 | 0.000 |  |
|  |  |  |  | **df** | 304 | 304 | 304 | 304 | 304 | 304 | 304 |  |
